# A Dosimetric Reappraisal of the Photobiomodulation Literature Assessing Animal Safety and Phototoxicity Evidence for Photobiomodulation in Oncology: Phantom Dangers

**DOI:** 10.3390/biomedicines14081859

**Published:** 2026-08-19

**Authors:** Mark Cronshaw, Steven Parker, James D. Carroll, Joel B. Epstein, Kinga Grzech-Leśniak, Michael R. Hamblin

**Affiliations:** 1 Leicester School of Pharmacy, De Montfort University, Leicester LE1 9BH, UK; thewholetooth@mail.com; 2 Thor Photomedicine, Chesham HP7 9FB, UK; 3 City of Hope National Medical Center, Duarte, CA 91010, USA; 4 Cedars-Sinai Medical System, Los Angeles, CA 90048, USA; 5 Laser Laboratory, Department of Integrated Dentistry, Faculty of Dentistry, Wrocław Medical University, 50-367 Wrocław, Poland; 6 Laser Research Centre, University of Johannesburg, Johannesburg 2092, South Africa

**Keywords:** photobiomodulation, oncology safety, dosimetry, beam profile, Gaussian beam, irradiance, fluence, Arrhenius framework, photothermal, immunodeficient model

## Abstract

Five studies are frequently cited as experimental evidence that photobiomodulation (PBM) may stimulate tumour growth or produce phototoxicity, contributing to a documented barrier to clinical adoption in oncology. We reappraise these studies against their own reported dosimetry. None characterised the spatial power distribution of its beam. Each was assessed for arithmetic consistency, beam profile, device category, and biological-model appropriateness, and re-examined against the Arrhenius framework for thermal damage. In four of the five, the exposures delivered lay outside the therapeutic photobiological window on the authors’ own stated parameters: surface temperatures of 55 °C and, in places, 71 °C, with injury the original authors describe as coagulation and necrosis extending to paralysis and death; irradiances approximately 25 times those used clinically; and, in one case, parameters the authors themselves describe as high. The fifth, a xenograft study, used a dose within the therapeutic range, but its immunodeficient host cannot express the immune-mediated response at issue. A sixth study, from the same group and using the same device as one of the five but operated at a therapeutic irradiance, is included as an internal control and reported therapeutic benefit. Injury in these regimes is best understood as oxygen-dependent, with temperature acting as a sensitising variable and as a marker of the regime rather than as the proximate cause, a reading supported by the original authors’ own helium-substitution, cooling and heated-probe controls. We outline a two-tier mechanistic framework, in which correctly dosed PBM may engage systemic anti-tumour immunity, as a hypothesis for prospective testing rather than as a finding of this analysis. None of these studies provides dosimetrically secure evidence of a hazard from therapeutic-range PBM; equally, the absence of such evidence is not a demonstration of safety. Beam-profile reporting should become standard in PBM oncology research.

## 1. Introduction

Photobiomodulation (PBM) therapy has achieved regulatory approval and guideline endorsement for the prevention and treatment of oral mucositis in patients receiving chemotherapy and/or head and neck radiotherapy [1,2,3]. Despite this, its adoption in oncological supportive care remains incomplete. A 2023 survey of MASCC/ISOO member clinicians identified concern over malignant transformation or accelerated tumour recurrence as a documented barrier to adoption [4]. This concern is not without a mechanistic rationale: in a review of PBM’s biological mechanisms, Sonis et al. observed that a number of the intracellular pathways activated by PBM, overlap with those implicated in tumour proliferation, invasion, angiogenesis, metastasis, and resistance to cancer therapy, and on that basis recommended that PBM not be applied directly over a tumour or a potentially malignant field [5].

For as long as PBM was commonly delivered as a focused beam applied to a defined site, this concern could be mitigated by a simple geometric precaution—directing the light away from the tumour. The modality, however, has changed. As Hamblin, Nelson, and Strahan observe, the increasing use of large-area light-emitting diode arrays and whole-body light-bed systems means that targeted avoidance of a lesion is no longer straightforward, and the question of whether these higher-power, large-field devices are safe in patients with cancer—or in otherwise healthy users harbouring an undiagnosed malignant or pre-malignant lesion—remains unresolved [6]. The safety question has, in effect, migrated towards the high-energy, large-aperture devices: precisely the class of system required to deliver therapeutic doses to tissue at volume and depth in oncological applications. A recent systematic review of PBM oncological safety frames the matter in the same terms, attributing the absence of a clear verdict to the interacting variables of wavelength, fluence and total delivered energy [7].

The experimental evidence underpinning this caution is mostly concentrated at the high-energy boundary. The small number of in vivo animal studies reporting tumour-growth stimulation by PBM did so at supra-therapeutic exposures—Frigo et al., for example, observed accelerated melanoma growth only at a high-power density (2.5 W/cm^2^) and high cumulative fluence (1050 J/cm^2^), and not at a lower dose [8]. A similar concern can be seen in phototoxicity literature, in which the same high-irradiance regime is reported to produce direct tissue injury; this experimental characterisation of near-infrared laser phototoxicity by Khan, Tang, and Arany is examined in detail here [9]. In contrast to both these concerns, the prevailing consensus that PBM is non-tumourigenic is itself explicitly conditional on the use of therapeutic parameters—characteristically low irradiance and single-digit fluences [10]. The conservatism surrounding high-power systems therefore rests on a body of evidence whose common and previously unexamined feature is the energy regime in which it was generated. We contend that this evidence warrants critical dosimetric reappraisal, and that—once the spatial distribution of beam power is properly accounted for—the apparent high-energy hazard is better explained as photothermal injury misinterpreted as a photobiological effect.

The photobiological window for PBM—an irradiance and fluence range within which mitochondrial photostimulation occurs without producing any photothermal injury—is well accepted [11]. Therapeutic PBM using correctly characterised parameters has been used in large numbers of oncology patients over more than two decades of clinical practice without any evidence of malignant transformation or accelerated tumour growth [10,12]. The studies producing contrary findings in animal models share a methodological feature that has not previously been systematically examined; namely, that none characterised the spatial power distribution of their laser beam. We propose that this omission is not incidental but is a determinant of the findings reported, and that correct dosimetric characterisation would reframe the biological interpretation of each study [13].

The studies most frequently cited in this context are summarised in a 2018 review by Hamblin, Nelson, and Strahan, which examined the evidence for and against PBM oncology safety and cited Sroka et al. (1999) [14], Sperandio et al. (2013) [15], Navratil and Kymplova (2002) [16], and Al Ghamdi et al. (2012) [17] alongside four of the five animal studies analysed in detail here. The wider list of concerns therefore comprises in vitro evidence of wavelength-dependent mitotic stimulation in tumour cell culture (Sroka et al.), Akt/mTOR pathway upregulation in dysplastic and oral cancer cell lines (Sperandio et al.), a narrative contraindications review (Navratil and Kymplova), and a cell proliferation review (Al Ghamdi et al.). None of these four additional studies used in vivo animal tumour models. All are in vitro or review publications and therefore do not directly model the clinical context of PBM in oncological supportive care. Critically, none of these studies reported beam characterisation data sufficient to determine whether the irradiance delivered at the cell surface corresponded to the therapeutic PBM window. The present study focuses its detailed dosimetric reappraisal on the five in vivo animal studies that are frequently cited as experimental evidence against PBM safety in cancer, because these carry the greatest weight in systematic reviews and guideline discussions.

A foundational principle of laser dosimetry is that the time-dependent spatial distribution of photon power across a beam profile determines the actual tissue exposure, not the average irradiance calculated by dividing total power by aperture area. All semiconductor diode lasers produce a Gaussian-profile beam unless specifically corrected by beam-shaping optics. For an unrectified Gaussian source, the peak irradiance at the beam centre substantially exceeds the average irradiance across the full aperture. The ratio depends on the specific beam parameters but is routinely 2× to 4× for the device classes used in the studies examined here.

Parker and Cronshaw previously demonstrated significant device-dependent power delivery losses in 38 calibrated dental diode laser units and established the importance of accurate radiometric characterisation and standardisation for PBM dosimetry [18]. Cronshaw et al. subsequently characterised photothermal effects of high-energy PBM in vitro across a range of wavelengths and power densities, confirming the importance of the spectral beam power profile across the beam area along with the corresponding irradiance as the critical biological parameter [19].

The present study applies a systematic dosimetric reappraisal to five studies cited as the primary evidence base for safety concerns about PBM in oncological contexts, together with a sixth study included as an internal control. We show that each used an unrectified Gaussian beam source and calculated the delivered dose from the aperture-averaged irradiance, systematically understating the irradiance actually delivered at the beam centre. In four of the five, the corrected exposure lay outside the photobiological PBM window on the authors’ own stated parameters, in the domain of photothermal tissue damage, and the biological findings are consistent with injury arising in that regime rather than with photobiomodulation. In the fifth, Rhee et al., the delivered dose lay within the therapeutic window, and the apparent tumour promotion is instead attributable to the use of an immunodeficient animal host. The internal control, a burn-wound-healing study by Khan et al. [20], used the same device as one of the five [9] but at a therapeutic irradiance and produced a beneficial healing outcome rather than the tissue damage seen at high intensity—a within-source demonstration that outcome tracks the delivered irradiance rather than the source itself.

The scope of this appraisal should be stated at the outset. It is a focused dosimetric and methodological critical appraisal of a small number of specific studies, not a systematic review, and it makes no claim to characterise the oncological safety of photobiomodulation as a whole. Its argument is negative and bounded: that the studies most frequently invoked as experimental evidence of oncological hazard rest on dosimetric foundations that do not support the inferences drawn from them. Demonstrating that a body of cited evidence is dosimetrically insecure is not equivalent to demonstrating safety, and the two should not be conflated. The subtitle “Phantom Dangers” refers specifically to the hazard claims attributed to these studies, and not to the question of PBM oncology safety in general, which remains open and requires prospective evidence.

## 2. Materials and Methods

### 2.1. Study Selection

This appraisal is a focused critical analysis of a small number of specific studies and is not a systematic review. The procedure by which those studies were identified is nonetheless set out here in full, so that it can be repeated, and its limitations are stated.

#### 2.1.1. Identification Criterion

The manuscript concerns studies that are frequently cited as experimental evidence of an oncological hazard associated with photobiomodulation. That criterion was operationalised as a count of appearances within a defined set of published reviews, systematic reviews and guideline documents addressing photobiomodulation safety in oncology, rather than as an assessment made by the author group. The six documents used for this count are listed in full in Supplementary Table S1a and comprise the systematic safety review of Bensadoun et al. (2020) [10]; the narrative safety review of Hamblin, Nelson, and Strahan (2018) [6]; the WALT position paper of Robijns et al. (2022) [21]; the commentary of Sonis et al. (2016) [5]; the MASCC/ISOO clinical-practice guideline of Zadik et al. (2019) [3]; and the umbrella review and national-society recommendations of Grzech-Leśniak et al. (2025) [2]. Each was read in full, and for every primary study the direction in which that document cites and classifies it was recorded.

Primary studies classified as showing tumour promotion in at least two of the traced documents and conducted in a tumour-bearing animal or carcinogenesis model were carried forward for appraisal. This selection rule is stated identically in the preamble to Supplementary Table S1. The operation is performed on publicly available documents and can be repeated independently; studies meeting the two-citation threshold but falling outside the animal-tier eligible universe are recorded in Supplementary Table S1b and addressed separately in Section 4.4.

#### 2.1.2. Context: A Screen of the Cancer-Safety Abstract Literature

To establish the denominator against which these studies should be read, the author group screened a curated collection of 328 abstracts addressing photobiomodulation and cancer, assembled by one of us (J.D.C.). Each record was assigned to a study-design tier, and each was assessed for the direction of the reported outcome. The tiers comprised in vitro (*n* = 146), human (*n* = 82), animal (*n* = 64), review (*n* = 12), out of scope (*n* = 13), and unclassified (*n* = 11).

Reported outcomes were categorised as promoting, inhibitory or protective, null, mixed or biphasic, or not determinable from the abstract. Across the collection, 35 records reported a promoting outcome, 132 an inhibitory or protective outcome, 59 a null outcome and 24 a mixed or biphasic outcome, with 96 not determinable from the abstract alone. The distribution across tiers is material: of 146 in vitro records, 26 reported promotion; of 64 animal records, 9 did; and of 82 human records, none did, with 30 of those 82 not determinable from the abstract.

Two limitations of this screen must be stated plainly, and it is reported here as context rather than as the identification method.

First, the collection is a curated private database rather than the output of a reproducible database search, and it cannot be regenerated by a reader from a stated search string. Second, the outcome tags on which the classification rests were assigned within the author group, including by an author with a declared commercial interest, and were cross-checked rather than independently duplicated. The screen therefore supports statements about the shape of the literature it contains; it does not establish that this literature is complete, and no conclusion in this manuscript rests on it alone.

#### 2.1.3. Eligibility and Assessment

Records carried forward were assessed against four criteria, defined before any recalculation was undertaken and applied uniformly:

1. **Arithmetic consistency** of the reported dosimetry, recomputed from the authors’ own stated values.
2. **Spatial power distribution**, whether measured, assumed, or unreported.
3. **Device category**, whether the source is engineered for photobiomodulation or for surgical application.
4. **Biological-model appropriateness**, with particular attention to host immune status, cell line provenance and the geometry of exposure.

Studies were excluded where the intervention was not photobiomodulation, where the record was a review rather than a primary study, or where the immune status of the host could not be determined from the published record. Every candidate, with its inclusion or exclusion decision and the rationale for that decision, is listed in Supplementary Table S1b.

#### 2.1.4. Studies Included from Outside the Screened Collection

Two of the studies analysed in Section 3 do not appear in the screened collection and were identified independently. Khan, Tang and Arany 2015 [9] is the source of the 45 °C surface-temperature threshold that has since been applied as a safety ceiling and is analysed here for that reason. Khan et al. 2021 [20] is not a hazard study at all; it is included as an internal control, being the same device and wavelength in the hands of the same group operated at a therapeutic irradiance and is analysed in Section 3.5 on that basis.

Their inclusion is therefore not attributable to the screen, and we record this so that the identification procedure is not represented as accounting for the whole of the selection.

### 2.2. Analytical Framework

Four analytical dimensions were applied to each study:

**Dimension 1—Arithmetic consistency:** Stated fluence values were verified by independent calculation from reported power, aperture area, and exposure time. Arithmetic errors were identified and the correct values substituted.

**Figure 1:**
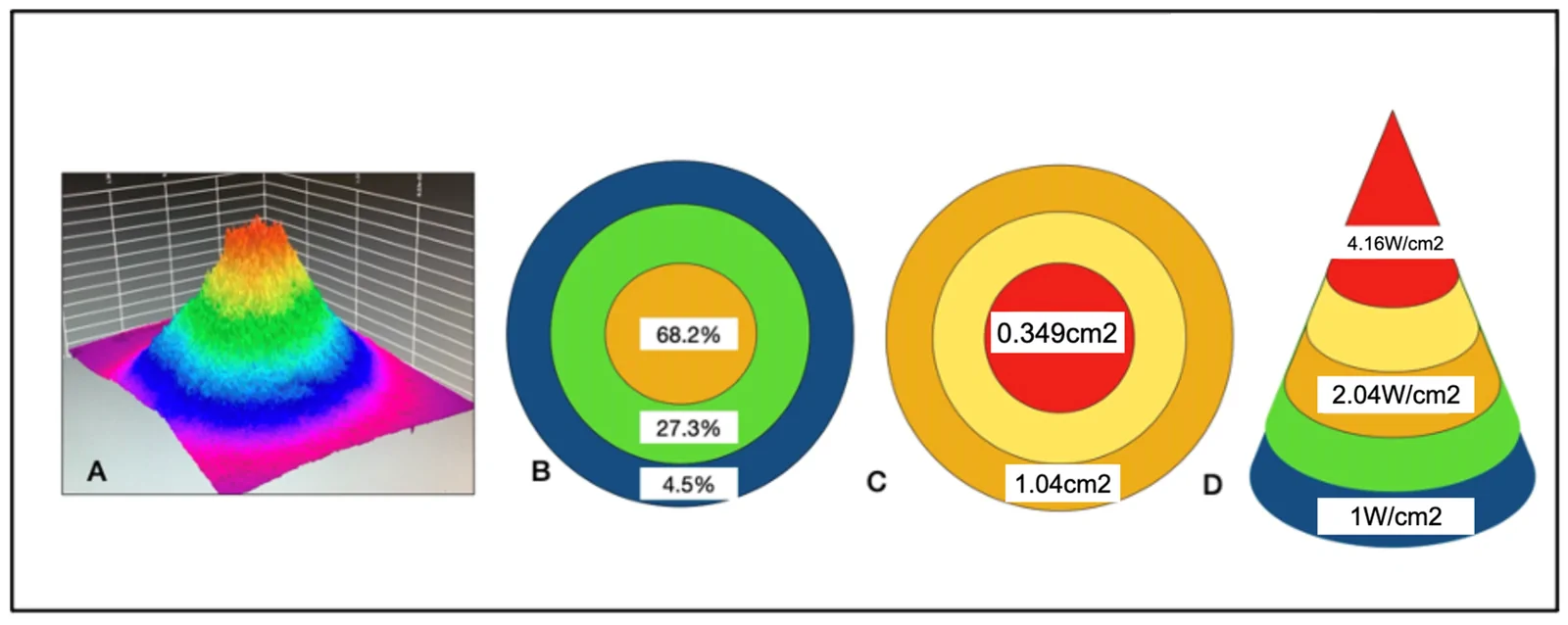
(**A**): 3D render of a standard Gaussian beam (Ophir Photonics beam profilometer, software: BeamGage 3.3.1). (**B**): Graphical breakdown of power distribution between outer, middle and central areas. (**C**): Central third power distribution continues to increase in the core of the beam. (**D**): Inner core area: power attains a peak in the centre of the beam. The example illustrates the effects of this progressive power peak towards the geometric apex of the beam. For a Gaussian beam of 2 cm in diameter, the area is ~3.14 cm^2^. Total power = 3.14 W, average power = 1 W/cm^2^. Tier 1 (central third, 1.047 cm^2^): 68% of 3.14 W = 2.14 W. Average irradiance = 2.14/1.047 = 2.04 W/cm^2^. Inner core area = 1.047/3 = 0.349 cm^2^. Average irradiance (inner core): 68% of 2.14 W = 1.45 W. 1.45/0.349 = 4.16 W/cm^2^. The effect on fluence results in a gradient with a central third peak: at 30 s exposure time the average fluence to the total area is highly misleading as there is a range according to outer, mid, or central concentric areas ranging in the case of the example above from ~0.7 J/cm ^2^ (outer third) to ~61 J/cm ^2^ (inner third) with a geometric peak in excess of 120 J/cm ^2^. Ref. Cronshaw et al. [19].

**Dimension 2—Gaussian beam correction:** For unrectified Gaussian diode laser sources, a multi-tier nested cascade was adopted. As described below (Figure 1), a 2 cm diameter beam has a central core occupying one third of the beam with an area of ~1.05 cm ^2^. This contains an average of 68% of the total power. A further second-tier area within the inner core contains approximately 46% of the total power in an area of 1.05/3 = 0.35 cm^2^. The method is described in detail in Cronshaw et al. [19].

**Dimension 3—Device category analysis:** The device category of each laser source was determined from manufacturer specifications and published characterisation data. Devices were classified as: (i) surgical/ablative lasers; (ii) therapeutic PBM devices; (iii) dental/ENT devices; or (iv) research devices. The clinical and biological implications of device category are discussed in Section 4.2.

**Dimension 4—Biological model critique:** The biological model used in each study was assessed for representativeness of the clinical context in which PBM is deployed, with particular attention to immune status of the animal host, cell line selection, and proximity to the irradiation source.

Throughout this appraisal, the admissibility of evidence is treated as purpose-relative. The claim under examination is that PBM stimulates tumour growth or induces phototoxicity in the clinical setting, which is a claim about an intact host and therefore depends on perfusion-mediated heat clearance, adaptive immunity, and stromal and vascular context. In vitro monolayer studies, in which cells are frequently irradiated in direct contact with the emitting aperture and without a convective or perfusive heat sink, cannot establish that claim in either direction; the limitation applies symmetrically to studies reporting hazard and to studies reporting safety. In vitro data are, however, admissible and probative for three distinct purposes on which the present analysis relies: characterisation of the delivered optical and thermal field, which is a physical rather than a clinical measurement; demonstration of irradiance- and wavelength-dependence under conditions in which temperature is directly measured rather than inferred; and the generation of mechanistic hypotheses to be tested subsequently in vivo, where such hypotheses are declared as such. Where in vitro work is cited in support of the present argument, it is cited for one of these three purposes and not as evidence of clinical outcome.

### 2.3. Thermal Calculations

For the Khan, Tang, and Arany 2015 study [9], surface temperatures are taken directly from the values the authors themselves measured and reported (located in their Supplementary Materials: Table S1; Supplementary Figures S1 and S8a; Supplementary Video S1) rather than estimated from a thermal model. No peak tissue temperature was back-calculated from fluence in the present analysis.

### 2.4. Arrhenius Thermal Damage Analysis—Methodology

Thermal damage to biological tissue follows the well-established Arrhenius relationship between temperature and exposure duration. The temperature threshold at which sustained thermal injury becomes appreciable is around 45 °C over hours of exposure; shorter exposures require correspondingly higher temperatures to produce equivalent damage. The Henriques–Moritz parameters (Henriques & Moritz 1947, as tabulated in Welch & van Gemert 1995) remain the canonical reference for skin damage modelling [22,23].

The Arrhenius equation is the standard engineering tool for calculating whether a given temperature sustained for a given time causes irreversible biological damage. It produces a single dimensionless number, Ω (omega). When Ω reaches 1, irreversible tissue damage has occurred. The relationship between temperature and Ω is exponential: a small increase in temperature produces a very large increase in Ω. The Henriques-Moritz parameters used in this analysis (A = 3.1 × 10^98^ s^−1^; Ea = 6.27 × 10^5^ J/mol; R = 8.314 J/mol·K) are those incorporated into ISO laser safety standards.

### 2.5. In Vitro Well-Plate Beam/Aperture Geometry Methodology

For in vitro studies in which the laser spot exceeded the well floor area, the beam/well geometric ratio is reported alongside the standard Gaussian cascade correction. Stated fluence at the cell surface is recalculated by multiplying the nominal fluence (calculated across the laser spot area) by the ratio of beam area to well area, capturing the effective concentration of beam-centre energy onto the well floor.

### 2.6. Radiometric Terms and the Relationship Between Irradiance and Fluence

Fluence, or radiant exposure, is the time integral of irradiance. The two are therefore not independent quantities and cannot meaningfully be placed in competition: any statement that one matters and the other does not is a statement about which is being held constant. They are nonetheless not interchangeable, because neither the thermal nor the photobiological response to light obeys reciprocity.

**The thermal response.** Tissue temperature during an exposure is set by the balance between the rate at which energy is deposited, which is proportional to irradiance and to the absorption coefficient of the tissue at the wavelength used, and the rate at which it is removed by conduction and perfusion. Where deposition is slower than clearance, temperature rises to a plateau and remains there; the Arrhenius damage integral accumulates negligibly however long the exposure continues, and total delivered fluence is in this regime almost irrelevant to thermal outcome. Where deposition outruns clearance, temperature rises progressively for as long as irradiation continues, and because the damage integral is exponential in temperature, it then accumulates rapidly. Two exposures delivering identical fluence at different irradiances can therefore produce entirely different thermal outcomes, and the Arrhenius formalism captures this precisely because it integrates over the temperature history rather than over delivered energy.

**The photobiological response.** Departures from the Bunsen–Roscoe reciprocity law are well documented in photobiomodulation and underlie the biphasic dose response. Lanzafame et al. [24] demonstrated this directly in vivo, holding radiant exposure constant at 5 J/cm^2^ per day in a murine pressure ulcer model while varying irradiance against exposure time across a matrix of combinations, and obtaining photostimulation at some combinations and not at others. Total dose alone did not determine the result.

**The formulation applied here** follows in two stages. Irradiance determines which domain an exposure occupies, photobiological or photothermal. Within a domain, fluence then describes the dose. Neither quantity is sufficient alone, and both must be reported together with exposure time, beam spatial power distribution, and the geometry of delivery, since none of these can be recovered from the others.

Three exposures analysed in this manuscript illustrate the two stages, and the second and third are the more instructive because they come from within the criticised literature.

Kalampouka et al. delivered an irradiance of 0.05 mW/cm^2^ sustained over 20 min, reaching a therapeutic-window fluence by way of duration rather than intensity. An optical temperature probe placed in the well confirmed that no thermal change occurred at any point. At this irradiance, no photothermal mechanism is available, and the observed effect was cancer-selective growth arrest rather than stimulation [25].

The two studies of Khan et al. examined in Section 3.4 and Section 3.5 hold the source constant and vary the irradiance. The same 810 nm diode laser, used by the same group, produced phototoxic injury at 1 W/cm^2^ and therapeutic benefit at 70 mW/cm^2^. This is the first stage in isolation: the domain moved because the delivery rate moved, while the device, the wavelength and the optical train did not.

The two studies by Frigo et al. examined in Section 3.2 do the converse, holding irradiance constant at 2.5 W/cm^2^ and varying only exposure time across 60, 180, and 420 s [8,26]. No effect was observed at 60 s and dose-dependent tumour promotion at the two longer exposures. This is the second stage in isolation, and it is the reason the formulation requires both parts: once an exposure sits in a regime where energy is being deposited faster than it can be cleared, duration determines whether enough accumulates to produce injury. Fluence is not irrelevant; it becomes decisive once irradiance has placed the exposure in a domain where accumulation can occur.

## 3. Dosimetric Reappraisal of the Individual Studies

### 3.1. Rhee et al. 2016 [27]

#### 3.1.1. Reported Data

Rhee et al. irradiated human anaplastic thyroid carcinoma (FRO) xenografts established in immunodeficient nude mice. The stated parameters were 650 nm, an irradiance of 100 mW/cm^2^, a spot area of 0.02 cm^2^, and a single exposure delivered in two dose groups: 15 J/cm^2^ over 150 s and 30 J/cm^2^ over 300 s. The authors reported increased tumour proliferation and angiogenesis in the irradiated groups, accompanied by decreased transforming growth factor-β1 and increased Akt and hypoxia-inducible factor-1α [27].

No spatial power distribution was reported, and no beam profile measurement is described.

#### 3.1.2. Verifiable Recalculation

The reported dosimetry is internally consistent. At 100 mW/cm^2^, the stated fluences follow directly from the stated exposure times (0.1 W/cm^2^ × 150 s = 15 J/cm^2^; 0.1 W/cm^2^ × 300 s = 30 J/cm^2^), and the corresponding delivered energies across the 0.02 cm^2^ aperture (0.3 J and 0.6 J) are self-consistent with both.

We record here, because it bears on the character of this reappraisal as a whole, that the arithmetic requires no correction in any of the five studies examined. Where their stated values can be recomputed, they reconcile. The criticism advanced in this manuscript is not of the authors’ arithmetic but of what their reported parameters omit, and of the exposure regimes those parameters describe.

Applying the beam-profile estimate described in Section 2.2 [28] to an unrectified Gaussian source of this aperture yields an estimated central irradiance in the region of 200–400 mW/cm^2^. Both the reported spatial average and the estimated central value lie within, or close to, the therapeutic photobiomodulation window. Neither lies in the photothermal or surgical range. On the Arrhenius framework applied in Section 2.3, no temperature history capable of accumulating a meaningful damage integral arises at these irradiances in perfused tissue.

The reported effect in this study therefore cannot be attributed to a dosimetric or photothermal artefact, and we do not argue that it can.

#### 3.1.3. Original Authors’ Interpretation

The authors interpreted their findings as evidence that low-level laser therapy promotes aggressive proliferation and angiogenesis in anaplastic thyroid carcinoma, mediated by suppression of TGF-β1 and upregulation of the Akt/HIF-1α axis, and concluded that caution is warranted in applying photobiomodulation to patients with malignancy.

#### 3.1.4. Alternative Interpretation Offered Here

We accept the dosimetry and question the inference drawn from the model.

The nude mouse is athymic and lacks functional T lymphocytes. The systemic anti-tumour activity proposed for correctly dosed photobiomodulation is immune-mediated, and an animal without adaptive immunity cannot express it. What such a host can express is the local component of the response—proliferative and angiogenic signalling in the irradiated field—with nothing acting in opposition. On this reading, the study measures one half of a two-sided process and reports the result as though it were the whole.

This is a limitation of scope rather than a fault of execution. The study is competent within its own terms; it is the extrapolation from an athymic host to an immunocompetent patient that does not follow.

It is worth stating why this study is examined at all, given that its host model is the reason its finding is set aside. Rhee et al. represent the strongest prima facie case for hazard in the animal literature, precisely because the dose is unobjectionable. A reappraisal that excluded it on model grounds without analysis would be open to the charge of setting aside the one study whose exposure it could not place outside the therapeutic window. It is retained and addressed for that reason.

#### 3.1.5. Degree of Certainty

The elements of this reappraisal are graded in Table 1 according to whether each is verified by the authors’ own reported values, plausible on a stated modelling assumption, or speculative.

#### 3.1.6. Principal Limitation of This Reanalysis

Our reading generates a prediction—that the same dose delivered to an immunocompetent host would not produce tumour promotion—which this study was not designed to test and which we cannot test from the published record. We do not claim that it does. The beam profile of the device was not available for characterisation, so the central irradiance estimate rests on the assumption set out in Section 2.2 rather than on measurement. And the interpretation offered here does not exclude the possibility that the reported signalling changes would occur in an immunocompetent animal as well, merely that they would there be met by an opposing response.

### 3.2. Frigo et al. 2009 and 2018 [8,26]

These two studies are treated together. They share a device, a wavelength, a delivery geometry, an animal model and a research group, and the latter study extends the dose range of the earlier one. Read as a pair, they constitute a three-point dose-response at fixed irradiance, which neither provides alone.

#### 3.2.1. Reported Data

**Frigo et al. 2009 [8].** Murine B16F10 melanoma cells were irradiated in vitro, and B16F10 tumours in male BALB/c mice in vivo, using a 660 nm InGaAlP diode laser with an unrectified Gaussian beam (Quasar Medical-Dentoflex) at 50 mW across a 2 mm^2^ spot, giving a stated irradiance of 2.5 W/cm^2^. Two in vivo dose groups were used, 150 J/cm^2^ and 1050 J/cm^2^ per session, delivered over three sessions. Tumour promotion was reported in the higher dose group only [8]. In the in vitro arm, cells were irradiated in direct contact with the emitting aperture.

**Frigo et al. 2018 [26].** The same group repeated the in vivo design with the same device, wavelength, power, spot area, and irradiance, in the same model, adding a third dose arm and changing the principal endpoints. Four groups were used: control, 3 J, 9 J, and 21 J, delivered daily over three days, with outcomes assessed as stromal collagen deposition (picrosirius red), blood vessel density (Verhoeff) and tumour volume. Significant dose-dependent increases were reported in the 9 J and 21 J groups. The 3 J group did not differ from control.

Neither study reports tissue thermometry. Neither reports a spatial power distribution.

This second study [26] is cited as evidence of oncological hazard in Bensadoun et al. 2020 [10] and therefore falls within the identification criterion set out in Section 2.1.

#### 3.2.2. Verifiable Recalculation

The stated parameters are internally consistent across both studies. A 50 mW output across a 2 mm^2^ (0.02 cm^2^) spot gives 2.5 W/cm^2^. The 2018 energies convert directly to the 2009 fluences: 3 J, 9 J, and 21 J across 0.02 cm^2^ correspond to 150, 450, and 1050 J/cm^2^, and at 50 mW to exposures of 60, 180 and 420 s, respectively. The two studies therefore describe the same delivery at three durations, with 2018 supplying the intermediate arm absent from 2009.

Three consequences follow arithmetically.

The stated irradiance of 2.5 W/cm^2^ is approximately 25 times the irradiance conventionally applied in therapeutic photobiomodulation. This holds before any consideration of beam profile.

The cumulative in vivo fluences run to 450 J/cm^2^ in the low-dose group and 3150 J/cm^2^ in the high-dose group across three sessions, one to three orders of magnitude above the few joules per square centimetre per session typical of clinical protocols.

Most usefully, the combined dose-response places a threshold between 150 and 450 J/cm^2^ at constant irradiance. Exposure duration alone distinguishes the harmless arm from the promoting arms. This is a direct empirical instance of the relationship set out in Section 2.6: irradiance determines which domain applies, and within a domain where energy is being deposited faster than it can be cleared, duration then determines whether enough accumulates to matter.

To these may be added two observations about the delivery geometry. A 2 mm^2^ spot corresponds to a diameter of approximately 1.6 mm, and an unrectified diode source at this aperture concentrates power strongly towards the axis, so the tissue on the beam axis received more than 2.5 W/cm^2^ by an amount that was not measured. And B16F10 is a heavily melanised line; melanin absorption at 660 nm is high, so absorbed energy density in this target substantially exceeds that in unpigmented tissue at the same incident irradiance.

#### 3.2.3. Original Authors’ Interpretation

The authors of both studies interpreted their findings as evidence that laser phototherapy can promote melanoma growth, the 2009 study reporting this at the greater of two doses and the 2018 study reporting dose-dependent increases in tumour volume together with stromal collagen and vascular changes at the two higher doses.

#### 3.2.4. Alternative Interpretation Offered Here

We accept the findings and dispute the range over which they generalise.

The host is immunocompetent, and the model is allogeneic, so no objection arises on either count; these are, on the face of it, the most relevant animal studies in the worry list. What removes them from bearing on therapeutic photobiomodulation is the irradiance. At 2.5 W/cm^2^ on a melanin-rich target with a 1.6 mm spot and no active cooling, energy is being deposited far faster than perfusion can clear it, and the exposure lies outside the photobiological window before duration is considered at all.

The dose-response the two studies jointly describe is what makes the pair informative rather than merely negative. Holding irradiance constant and varying only time, no effect is seen at 60 s, and dose-dependent promotion appears at 180 s and 420 s. A threshold that appears with accumulated exposure at fixed delivery rate is the signature of an accumulating process rather than of a photobiological dose-response, and it is the behaviour the Arrhenius formulation in Section 2.3 predicts. On this reading, the 2018 study does not extend the evidence of hazard; it locates the boundary of the regime in which hazard appears, and places the harmless arm nearest to, though still well above, the clinical range.

We note that these authors did what the wider literature has generally not done, which is to vary dose systematically within a single design. The criticism here is of the range selected, not of the conduct of the work.

#### 3.2.5. Degree of Certainty

Table 2 grades each element of this reappraisal according to whether it is verified by the authors’ own reported values, plausible on a stated modelling assumption, or speculative.

#### 3.2.6. Principal Limitation of This Reanalysis

Neither study reports tissue temperature, so the thermal reading of the threshold is inferred from irradiance and duration rather than measured, and an alternative account in which the threshold reflects a genuinely biphasic photobiological dose-response cannot be excluded from the published data. Neither device was characterised, so the beam-profile estimate rests on the assumption set out in Section 2.2. The 2018 endpoints include stromal and vascular measures whose relationship to tumour behaviour is less direct than volume, and we have not attempted to weigh them separately. And while the 150 J/cm^2^ arm is the closest thing in this literature to a negative control at sub-threshold dose, it remains an order of magnitude above clinical exposure, so it does not by itself demonstrate safety within the therapeutic range.

### 3.3. De C Monteiro et al. 2011 and 2013 [29,30]

#### 3.3.1. Reported Data

De C Monteiro et al. irradiated DMBA-induced oral squamous cell carcinoma in the golden Syrian hamster cheek pouch using a 660 nm GaAlAs diode laser (Laserpulse, Ibramed, Brazil) at a stated output of 30 mW across a stated area of 0.07 cm^2^, giving an irradiance of approximately 424 mW/cm^2^ and a fluence of 56.4 J/cm^2^ per session. Output was verified with a Thorlabs PM30 power meter. Groups comprised five animals. The authors reported laser-associated progression of carcinoma severity, with a reported *p*-value of 0.04 [29].

In their discussion, the authors state that the irradiation parameters used may be considered high, and that a lower dose in the carcinogen-plus-laser group might have been more appropriate. They further note the limited statistical power of the design and the confounding contribution of the carcinogen administration protocol.

No spatial power distribution was measured or reported, and no tissue temperature was recorded at any point.

A companion report from the same laboratory extends the same experiment. De C Monteiro et al. 2013 [30] used the identical DMBA hamster cheek pouch model, the identical 660 nm source, the identical 3 mm spot of 0.07 cm^2^, the identical 133 s continuous exposure and the identical every-other-day schedule over four weeks, with groups of five animals and the same Thorlabs PM30 power meter. The single parameter that differs is average power: 50 mW rather than 30 mW, giving 714.2 mW/cm^2^ and 95 J/cm^2^ per session. The 2013 study additionally included imiquimod and combined imiquimod-plus-laser arms.

The outcome in the laser-only arm ran in the same direction as the 2011 report. Poorly differentiated carcinoma was present in two of five irradiated animals and in none of the 10 control animals, and well-differentiated carcinoma was significantly more frequent in the controls at a reported *p*-value of 0.04. The authors declined to claim significance for the poorly differentiated comparison itself. The two papers are therefore not a promoting result and a null one, as they are sometimes tabulated, but two points on a single dose escalation. The 2013 report is not classified as evidence of tumour promotion in any of the documents traced in Section 2.1.1 and is therefore not one of the five studies identified by that procedure. It is appraised here because it is the same experiment at a higher exposure, and because the two reports are more informative together than either is alone.

The 2013 report also records, in its parameter table, a field headed beam profile measurement, the entry for which is “round”. The authors further describe the hamster cheek pouch as a site of immune tolerance in which lymphatic drainage is practically absent and dendritic cells are few.

#### 3.3.2. Verifiable Recalculation

The arithmetic is internally consistent: 30 mW distributed across 0.07 cm^2^ yields approximately 428 mW/cm^2^, in agreement with the authors’ stated 424 mW/cm^2^.

Two observations follow, both derivable from the published record.

The stated irradiance is approximately four times the irradiance conventionally applied in therapeutic photobiomodulation, and the per-session fluence exceeds by roughly an order of magnitude the few joules per square centimetre typical of clinical protocols. This comparison requires no modelling; it is a comparison of the authors’ own stated values against published therapeutic ranges.

The Thorlabs PM30 integrates total incident power over the detector and does not resolve spatial distribution. The stated 0.07 cm^2^ is an aperture area rather than a measured beam width. The reported irradiance is therefore an aperture average, and for any non-uniform profile the irradiance on the beam axis necessarily exceeds it. The magnitude of that excess cannot be determined, since the device was not characterised, but on the estimate applied in Section 2.2 a central irradiance in the region of 0.8–1.7 W/cm^2^ would follow.

The companion study permits one further observation that requires no modelling. Because every delivery parameter except average power is held constant between the two reports, they constitute a two-point dose escalation performed within a single laboratory in a single model: 424 mW/cm^2^ and 56.4 J/cm^2^ at 30 mW, against 714.2 mW/cm^2^ and 95 J/cm^2^ at 50 mW. Set against the MASCC/ISOO recommended protocol for head and neck chemoradiotherapy, which specifies 660 nm at 417 mW/cm^2^ for 10 s per site to a fluence of 4.2 J/cm^2^, the lower point matches the recommended irradiance but delivers it for 13 times as long to 13 times the fluence, and the upper point exceeds the recommended irradiance by a factor of 1.7 and the recommended fluence by a factor of 23. Neither point tests a therapeutic exposure.

The beam profile field in the 2013 parameter table is worth recording precisely because the report is otherwise conscientious. Every reported quantity in it reconciles, the power meter is named, and the aperture area is given as a measurement. The profile entry nevertheless describes the shape of the aperture rather than the distribution of irradiance within it. A round spot may carry any radial distribution whatever, and the quantity that determines the exposure at the beam axis is not recorded.

We note separately that the reported statistical result is fragile. With five animals per group, a *p* value of 0.04, and the effect driven by two animals exhibiting poorly differentiated carcinoma, reclassification of a single animal would remove significance. This is an observation about the published design, not a criticism of its conduct.

#### 3.3.3. Original Authors’ Interpretation

The authors interpreted their findings as evidence that laser phototherapy at the parameters used influenced the outcome of chemically induced oral carcinogenesis unfavourably, while themselves qualifying the result on grounds of dose, statistical power and the carcinogen protocol.

#### 3.3.4. Alternative Interpretation Offered Here

This study does not dispose of itself on the grounds available for the others, and we state that plainly. The host is immunocompetent, so the objection applied to Rhee et al. does not arise in the form it takes there, although the authors of the companion study describe the cheek pouch itself as an immune-tolerant compartment with practically absent lymphatic drainage, which qualifies the model rather than the animal. The device is a dedicated photobiomodulation instrument operating at 30 mW, so the device-category objection applied to Khan et al. does not arise in the form it takes there. It does not follow that the beam was homogenised, for the reasons given at Section 4.2; marketing category and beam correction are independent, and the profile of this instrument is neither reported by the authors nor known to us. No surface temperature approaching a coagulation threshold is recorded, because no temperature was recorded at all.

Our reading rests on the convergence of three separate observations rather than on any one of them.

The exposure sits above the therapeutic range on the authors’ own figures, by a factor of roughly four in irradiance and an order of magnitude in per-session fluence. The reported irradiance is an aperture average obtained with an instrument that cannot resolve distribution, so the tissue on the beam axis received more than the stated figure by an amount that was neither measured nor corrected for. And the authors themselves regarded their parameters as excessive and their design as underpowered.

Taken together, these support the reading that the study characterises the behaviour of light delivered above the therapeutic window rather than within it. They do not establish a thermal mechanism, and we do not claim one: without thermometry, the contribution of temperature to the observed progression is unknown.

The dose escalation between the two reports should be interpreted with care, because it does not discriminate between the two readings available here. Both a supra-therapeutic delivery artefact and a genuine dose-dependent biological effect predict a larger effect at the higher exposure, and the escalation is consistent with either. What it does establish is narrower and not in dispute: across both reports, the exposure tested lies outside the therapeutic window on every axis, and no exposure within that window was examined in either.

#### 3.3.5. Degree of Certainty

The elements of this reappraisal are graded in Table 3 on the same basis, distinguishing what is verified by the authors’ own reported values from what rests on a stated modelling assumption.

#### 3.3.6. Principal Limitation of This Reanalysis

This is the study that our reanalysis disposes of least securely, and it should be read in that light. The host model is appropriate, the device is a therapeutic instrument, and the absence of thermometry means that no measured temperature is available either to support or to exclude a thermal contribution. Our reading rests on three moderate lines of argument that converge rather than on a single decisive one, and a reader who declines to weigh the authors’ own qualification of their parameters is left with a smaller case than the one we have made. We would also note that the alternative we favour, namely that the finding reflects supra-therapeutic delivery, is not the only reading available: a genuine dose-dependent effect at this irradiance in a carcinogen-primed epithelium cannot be excluded from the published data and would be a legitimate subject for a properly powered study with thermometry and characterised beam delivery. If such an effect were real, it would bear on the use of high cumulative doses over pre-malignant tissue specifically, not on therapeutic photobiomodulation delivered at clinical doses, which is the exposure this study does not test.

The addition of the 2013 companion report strengthens the description of the exposure without strengthening the inference drawn from it. It confirms that this laboratory worked consistently above the therapeutic window and that the direction of the reported effect was reproduced at a higher dose, but a reproduced effect at a higher dose is equally consistent with the reading we do not favour. This section should accordingly be read as establishing what these two studies did not test, rather than as establishing what a therapeutic exposure would have done.

### 3.4. Khan, Tang and Arany 2015 [9]—ATF-4/Phototoxicity Paper

#### 3.4.1. Reported Data

Khan, Tang, and Arany investigated near-infrared laser phototoxicity in murine and cellular models using an 810 nm GaAlAs diode laser (AMD Lasers, West Jordan, UT, USA) at a 2 cm working distance and 2 cm spot diameter, with a stated average irradiance of 1 W/cm^2^ and in vivo exposures of 10–30 s [9].

In vivo, phototoxic skin damage was defined by surface temperature, with onset reported at 45 °C (their Supplementary Table S1). The authors measured surface temperatures reaching their 55 °C target and, in places, 71 °C (their Supplementary Figure S8a). Their own dosimetry assumes a Gaussian beam distribution (their Supplementary Table S4), consistent with the rotationally symmetric profile visible in their thermal-camera image (their Figure 1). In the in vitro experiments, conducted in 96-well plates at a stated spot diameter of 3.8 cm, a phototoxic threshold of 27 J/cm^2^ over 300 s was reported.

The authors attributed the phototoxicity to ATF-4-mediated endoplasmic reticulum stress.

Four control conditions reported in the same study bear directly on mechanism and are set out here as reported data rather than as interpretation. A metal probe held at 45 °C produced no significant skin damage, while laser treatment reaching the same surface temperature produced a substantial mean damaged area (their Figure 1e). Laser treatment carried out at 4 °C did not generate phototoxicity. Replacing oxygen with helium in the medium abolished phototoxicity despite an unchanged rise in surface temperature. Pre-incubation of cells at 42 °C lowered the phototoxic threshold from 2 W to 1.5 W. In vivo, cryogen spray cooling of the treated area abolished both the damaged area and the erythema score following a 55 °C exposure, and pre-treatment with N-acetylcysteine reduced both. Cooling and N-acetylcysteine also reduced reactive oxygen species induction in vivo, measured with the ROSstar probe by in vivo imaging.

The authors further report, in their Supplementary Table S1, a graded scale of injury by surface temperature: no effect below 45 °C; mild oedema, erythema and a damaged area at 45 °C; prominent oedema, severe erythema and burn injury with involuntary limb extension between 45 and 55 °C; and above 55 °C, ulceration, contractures, paralysis, and death. Their Supplementary Figure S1 legend describes the corresponding histology above 55 °C as coagulation and necrosis of skin and connective tissue, muscle, bone marrow, and peripheral nerve, with degenerative changes in the spinal cord. Surface temperatures in the immunoblot series of their Supplementary Figure S8a extend to 71 °C. In addition, purified glutathione reductase and catalase lost activity following irradiation in strongly absorbing black-well plates but not in weakly absorbing clear-well plates (their Supplementary Figure S3h,i), with a corresponding shift of the cellular glutathione pool towards its oxidised form. Ames and plasmid-cleavage assays were negative.

No spatial power distribution was measured or reported.

#### 3.4.2. Verifiable Recalculation

The authors’ stated arithmetic is internally consistent, and we identify no error in it. Two geometric observations follow from their own reported values.

A prior observation about the in vivo exposures should be recorded, because it determines what can and cannot be recalculated from them. The Methods give a single figure of 21 J/cm^2^ at 1 W/cm^2^. Neither the exposure times stated in the text nor those shown in the authors’ own thermometry correspond to it. Their Supplementary Figure S1d–f plots surface temperature against time for the three in vivo conditions, and in each the labelled interval is the dwell at target temperature rather than the total irradiation: the traces begin near 27 °C and ramp for approximately 15, 25, and 11 seconds, respectively, before the labelled 10 or 30 s commences. The phototoxic condition on which the study turns, 45 °C for 30 s, therefore involved the order of 55 s of irradiation. The stated 21 J/cm^2^ corresponds to 21 s and matches no phototoxic condition.

We do not present this as an arithmetic error, because the delivered energy is not determinable in the first place. The beam was switched on and off through the plateau to hold the target temperature, so total elapsed time is an upper bound on exposure and the duty cycle is not reported. Nor were power and time held constant: the authors state that both were varied according to melanin score, and the 55 °C trace ramps more than twice as fast as the 45 °C trace, which implies a higher power. No single fluence describes these exposures, and none is recoverable from what is reported. This is the substitution of temperature for dose in its clearest form: the controlled variable was thermal, and the radiometric quantity that would permit comparison with any other study was neither held constant nor recorded.

**In vivo.** The authors assume a Gaussian distribution. For such a distribution, the power is concentrated towards the axis rather than distributed evenly across the aperture: on the framework applied in Section 2.2, the central third of the beam area carries approximately 68% of the total power, corresponding to roughly twice the nominal average, with the inner core approximately four times the average. Applied to the stated 1 W/cm^2^, this places the estimated centre-of-beam irradiance at or above the photothermal-damage threshold at 810 nm, with the inner core several-fold above it.

This estimate is not required for the central point, which rests on measurement rather than modelling. The authors recorded surface temperatures of 55 °C and, in places, 71 °C. At 71 °C, protein denaturation and coagulation occur on any published formulation of the Arrhenius damage integral. No back-calculation of peak temperature from fluence is performed here or needed: the temperatures associated with the observed injury are those the authors themselves measured.

**In vitro.** The authors’ Supplementary Table S4 reports paired conditions for the two plate formats used: 4.3 W at 14.5 cm with a 5.5 cm spot diameter for the 6-well plate and 2 W at 10 cm with a 3.8 cm spot diameter for the 96-well plate, both stated as 0.090 W/cm^2^ and, over 300 s, 27 J/cm^2^. Computed directly from the stated spot diameters, these powers give 0.181 and 0.176 W/cm^2^ respectively, so a correction of approximately one half has been applied in both cases. The table legend attributes this to power-meter detector surface coverage falling from 20% to 10% between the two working distances, incorporated on an assumed Gaussian distribution.

Two observations follow, and it should be stated clearly that the authors did address beam profile in their dosimetry rather than neglecting it.

First, the derivation of the correction is not given, and the relationship between measured power, detector coverage, and reported irradiance cannot be reconstructed from the published record. The reported figure is therefore not independently verifiable, though it is evidently the product of a deliberate correction rather than a simple division of power by aperture area.

Second, a 3.8 cm spot spans a substantial fraction of a 96-well plate, and the authors’ method states that the working distance was chosen so that the spot covered the treatment surface. Cells in wells near the beam axis and cells in wells towards the periphery therefore cannot have received the same irradiance, and a single stated value cannot describe both. This is a limitation on the precision with which the phototoxic threshold is localised rather than an error in the calculation, and we note it as such. The authors’ own coverage experiment, in which cytotoxicity increased as the proportion of the well surface covered with absorbing tape increased from one third to one half to full (their Supplementary Figure S3a), demonstrates that absorbed area is a determinant of outcome in this model and is consistent with this observation.

**Application technique.** The authors state that the probe was moved continuously above 55 °C to prevent excessive heating, and their supplementary video shows movement about a central axis rather than systematic traversal of a defined area. Beam movement is an effective mitigation of heat accumulation where it allows thermal relaxation between passes, but its effectiveness depends on the pattern and rate of traversal, and movement about a fixed point does not distribute energy in the way that scanning across an area does. This bears on the highest-temperature conditions only; the exposures defining the 45 °C threshold were delivered with a static beam.

#### 3.4.3. Original Authors’ Interpretation

The authors interpreted the observed phototoxicity as mediated by ATF-4-dependent endoplasmic reticulum stress, established 45 °C as a surface-temperature threshold for phototoxic injury, and proposed this threshold as a safety ceiling for near-infrared exposure.

#### 3.4.4. Alternative Interpretation Offered Here

Two separate claims are made, and they should not be conflated.

**On dosimetric regime.** The in vivo exposures produced measured surface temperatures well above the authors’ own 45 °C threshold and, at 71 °C, within the range at which coagulation occurs. Whatever the mechanism of injury, these exposures did not take place within the therapeutic photobiological window, and results obtained there do not characterise the behaviour of photobiomodulation delivered within it. This is the claim on which our argument depends, and it rests on the authors’ own thermometry.

It should be recorded that these authors, alone among the five, recognised the beam-profile problem and attempted to allow for it. The allowance was nonetheless smaller than the discrepancy it was intended to correct, and it was not accompanied by scanned delivery at the higher end of the dose range, where a static exposure permits thermal accumulation at the beam axis over the full irradiation time. The criticism made here is therefore one of degree rather than of omission, and it is the more instructive for that: a correction applied in good faith proved insufficient because the scale of the peak-to-average discrepancy was not appreciated.

On mechanism. The loss of catalase and glutathione reductase activity in the strongly-absorbing condition is oxidative rather than a thermal inactivation, and no fixed inactivation temperature can be attached to it. No temperature was recorded for the enzyme preparations; the 45 °C figure in this paper is its phototoxicity threshold rather than an enzyme inactivation point; and neither enzyme is thermally labile in the relevant range. Reported denaturation temperatures for the holo Cu/Zn-SOD1 enzyme vary with species, scan rate and buffer conditions, the unfolding being kinetically controlled and irreversible: values for the bovine enzyme cluster between approximately 89 and 104 °C [31,32], and the lowest human wild-type transitions reported anywhere in this literature lie at approximately 75 and 84 °C. We take the lowest of these figures rather than the highest, because the argument does not need the range and is stronger without it. Even on the most conservative reading available, the enzyme retains its fold some 30 degrees above the 45 °C threshold at issue. Glutathione reductase is likewise not thermally labile here: its activity in human erythrocytes is unaffected by heat stress across the physiological and mildly hyperthermic range [33].

The authors’ own controls indicate the correct reading. Helium substitution abolished phototoxicity while the temperature rose exactly as in the untreated arm, which places the effector on the oxidative side. A metal probe at 45 °C produced no damage, so heat alone at the stated threshold is insufficient. Cells at 4 °C were protected, and pre-incubation at 42 °C, harmless in itself, lowered the threshold from 2 W to 1.5 W. Taken together, these establish an oxygen-dependent mechanism in which temperature lowers the dose at which oxidative disabling of the scavenger enzymes occurs, and marks the regime within which it occurs, without being its proximate cause.

**On ATF-4.** We do not propose photothermal injury in place of endoplasmic reticulum stress, and the authors’ ATF-4 findings are not in dispute. ATF-4 translation is the convergence point of the integrated stress response, which is driven by four eIF2α kinases responding to distinct insults including oxidative stress and heat shock [34,35,36]; hyperthermia alone is sufficient to induce eIF2α phosphorylation followed by ATF-4 and CHOP. ATF-4 induction is therefore a general signature of cellular insult rather than of any particular initiating event. What is at issue is not whether ATF-4 was induced but what induced it, and on the evidence of the authors’ own controls that was an oxidative burden generated under absorbed light, sensitised by temperature.

#### 3.4.5. Degree of Certainty

Table 4 grades each element of this reappraisal, separating the quantities verified from the authors’ own thermometry and reported values from those which depend upon an assumed beam profile.

#### 3.4.6. Principal Limitation of This Reanalysis

The device was not available for characterisation, so the beam profile is assumed rather than measured and the 2–4× estimate should be read accordingly. The distribution of temperature across the irradiated field cannot be recovered from the published record, so we cannot state what proportion of the tissue experienced 71 °C rather than 55 °C. Our reading of the supplementary video is an interpretation of a recording, not a measurement of beam motion. The in vitro geometric analysis assumes standard 96-well dimensions and a centred beam, neither of which is independently confirmed. And the account offered here does not exclude a contribution from direct photochemical routes operating alongside the oxidative one; it holds only that the initiating event was not thermal denaturation of the scavenger enzymes.

### 3.5. Khan et al. 2021 [20]—The Same Device Within the Therapeutic Range

This study is not one of the five cited as evidence of hazard, and it is not analysed as such. It is included as an internal control: the same research group, using the same device and wavelength, operating at a therapeutic irradiance. Its evidential value lies in the comparison with Section 3.4, not in its own findings, which we do not dispute.

#### 3.5.1. Reported Data

Khan et al. used the same AMD 810 nm GaAlA diode laser employed in the 2015 phototoxicity study, in a burn wound healing model in immunocompetent C57BL/6 mice. The stated protocol was an average irradiance of 70 mW/cm^2^, an exposure of 30 s, delivered three times weekly. The authors reported accelerated healing, mediated through TGF-β1 signalling [20].

#### 3.5.2. Verifiable Recalculation

The stated parameters give a per-session radiant exposure of 2.1 J/cm^2^ (0.07 W/cm^2^ × 30 s), which lies within the range conventionally applied in therapeutic photobiomodulation.

The comparison with Section 3.4 is the point of the section. The same device, at the same wavelength, in the hands of the same group, was operated at 1 W/cm^2^ in the 2015 study and at 70 mW/cm^2^ here, a difference of approximately 14-fold in irradiance. Applying the beam-profile estimate of Section 2.2 to both leaves the 2021 exposure within the therapeutic window even at the beam centre, whereas the same correction places the 2015 exposure at or above the photothermal threshold. The device did not change; the domain did.

#### 3.5.3. Original Authors’ Interpretation

The authors interpreted their findings as demonstrating a therapeutic effect of photobiomodulation on burn wound healing mediated by TGF-β1.

#### 3.5.4. Interpretation Offered Here

We do not offer an alternative reading of this study. We accept it as reported and draw a single inference from the pair.

A source capable of producing phototoxic injury at 1 W/cm^2^ produced therapeutic benefit at 70 mW/cm^2^. Wavelength, device, optical train, working geometry, and research group were held constant across the two studies; irradiance was not. This is a within-source demonstration that the outcome of an exposure is determined by the rate at which energy is delivered rather than by the identity of the source or by total energy alone, and it is the empirical anchor for the formulation set out in Section 2.6.

It bears directly on how the 2015 study should be read. The phototoxicity reported there is not a property of near-infrared light, of 810 nm, or of that laser. It is a property of the irradiance at which that laser was operated in that experiment, as the same authors demonstrated six years later using the same instrument.

We would note that this comparison is available only because the group returned to the same device at a different setting. Very few sources in this literature have been characterised across their operating range in this way, which is why a single research group’s two papers can settle a question that the wider literature leaves open.

#### 3.5.5. Degree of Certainty

Table 5 grades the elements of this control comparison on the same basis as the preceding studies.

#### 3.5.6. Principal Limitation of This Reanalysis

The two studies differ in more than irradiance. The 2015 study examined phototoxicity in skin and in cell culture; the 2021 study examined healing in a burn wound model in a different mouse strain. The comparison therefore holds device, wavelength and research group constant but not indication, tissue state, or host, and it would be overstated to present it as a controlled dose-response. What it supports is narrower and still useful: that no property intrinsic to this source or this wavelength accounts for the 2015 findings, since the same source produced benefit when operated at a therapeutic irradiance. A formal demonstration would require the same model irradiated across a range of irradiances with thermometry, which neither study provides.

### 3.6. Synthesis of Dosimetric Findings Across the Reappraised Studies

The five studies examined share three compounding methodological features.

First, all five used unrectified Gaussian sources and calculated delivered fluence from total aperture area. For any spatially non-uniform distribution, the irradiance on the beam axis necessarily exceeds the aperture average, so the central irradiance in each case was understated. The direction of that error follows without assumption; its magnitude, estimated here at approximately two- to four-fold, rests on the model described in Section 2.2 and is presented as an estimate rather than a measurement.

Second, device category varies and matters. Khan et al. used a diode laser engineered for soft-tissue surgery, operated at 1 W/cm^2^ over a 2 cm spot. Frigo et al. and de C Monteiro et al. used devices dedicated to photobiomodulation but operated them at 2.5 W/cm^2^ and 424 mW/cm^2^, respectively, in the first case approximately 25 times and in the second approximately 4 times the irradiance conventionally applied in clinical protocols. A therapeutic device operated outside its therapeutic range does not thereby deliver therapy.

Third, biological-model appropriateness differs across the group and does not fall uniformly against the studies. Rhee et al. used an immunodeficient nude-mouse host without functional T lymphocytes, which cannot express an immune-mediated response. Frigo et al., by contrast, used immunocompetent BALB/c mice injected with B16F10 melanoma cells, which are syngeneic to C57BL/6 rather than to BALB/c. Although this is not a conventional pairing, Foerster et al. found B16F10 cells to be more metastatic in BALB/c than in syngeneic C57BL/6 hosts, attributed to higher natural killer cell activity in the latter, so the model is not straightforwardly permissive [37]. de C Monteiro et al. used an immunocompetent chemical-carcinogenesis model with parameters their own authors acknowledged as excessive.

Taken together, in four of the five studies the exposures delivered lay outside the therapeutic photobiological window on the authors’ own stated parameters, before any correction for beam profile is applied, and the biological findings are consistent with injury arising in that regime. The exception is Rhee et al., where the delivered dose lay within the therapeutic window; there it is the host model rather than the dosimetry that limits the inference, since the immunodeficient animal removes the adaptive immune response through which PBM is proposed to exert anti-tumour effects. The internal control examined in Section 3.5 completes the picture: the same source that produced phototoxic injury at 1 W/cm^2^ produced therapeutic benefit at 70 mW/cm^2^.

In none of these studies, therefore, is there dosimetrically secure evidence that therapeutic-range PBM delivered to an immunocompetent, clinically representative model stimulates tumour growth.

## 4. Discussion

**Why tissue temperature matters**

Skin and soft tissue respond predictably to heat. Below 40 °C, cells function normally. Between 40 °C and 45 °C, mild and reversible physiological changes begin, and the heat-shock response is engaged at 40–42 °C. At 45 °C, the Arrhenius damage integral begins to accumulate at a rate that depends strongly on the exposure duration; using Henriques–Moritz kinetics, the time to Ω = 1 at constant 45 °C is of the order of hours, well below the threshold for bulk tissue protein coagulation. The 45 °C value Khan et al. established in their Supplementary Figure S1 [9] as the onset of phototoxic skin damage is therefore consistent with a sub-coagulation regime, in which injury is mediated not by gross protein denaturation but by loss of antioxidant defence, discussed below. At 60 °C and above, tissue proteins coagulate rapidly, and the surface temperatures the authors recorded reached 71 °C (Khan et al., Supplementary Figure S8a)—the range used deliberately in surgical laser procedures to coagulate tissue.

**Oxidative inactivation of antioxidant enzymes, with temperature as a sensitising variable**

Supplementary data from Khan et al. provide the molecular mechanism for the observed biological findings, and it is oxidative rather than a direct photobiological interaction. Their Supplementary Figure S3 [9] shows (panels h and i) that purified catalase and glutathione reductase lose activity following the laser dose in strongly absorbing black-well conditions but not in the cooler clear-well condition. This is not thermal denaturation: glutathione reductase activity in human erythrocytes is unaffected by heat stress across the physiological and mildly hyperthermic range [33], and the holo Cu/Zn-SOD1 enzyme retains its fold at temperatures far above the range at issue, the most conservative human values reported lying at approximately 75 and 84 °C [31,32]. Neither enzyme is denatured at the temperatures relevant to the phototoxicity threshold. The differentiating factor between the two well conditions is the quantity of absorbed light and the reactive oxygen species (ROS) it generates, with temperature acting as a sensitising variable rather than as the agent of enzyme loss—a reading the authors’ own controls support, since replacing oxygen with helium abolished phototoxicity while surface temperature rose unchanged. Once the peroxide-clearing enzymes are oxidatively disabled, uncontrolled ROS accumulation drives lipid peroxidation and the generation of secondary nitrosyl species. The ATF-4 and ER-stress response that Khan et al. identified is a downstream consequence of this oxidative cascade in cells at the beam margin that survive long enough to mount a stress response, rather than evidence of a direct photobiological effect. (This mechanism is developed further, with the role of the constitutive antioxidant deficit of the tumour cell, in Section 4.6).

Khan et al. interpret their findings as a photobiological ATF-4/ER-stress response leading to apoptosis. At the surface temperatures the authors recorded, however—up to 71 °C (Khan et al.’s Supplementary Figure S8a)—the central irradiated tissue lies in the range of frank thermal coagulation and necrosis, consistent with the coagulation and necrosis described in the authors’ own Supplementary Figure S1 legend. The ATF-4 findings are most plausibly read as genuine stress-response signalling in cells at the cooler beam margins, superimposed on a core of oxidative and, at the highest temperatures, coagulative injury. The distinction carries an immunological consequence, since necrosis releases pro-inflammatory damage-associated molecular patterns whereas apoptosis does not. Khan’s negative Ames and plasmid-cleavage data are consistent with an absence of direct genotoxicity under either reading.

Khan et al. identify 45 °C as the threshold for the phototoxicity they observe in mouse skin and propose a mechanism involving photochemical generation of ROS with secondary inhibition of catalase and glutathione peroxidase [9]. However, several aspects of their own data point more directly to a thermal mechanism with downstream ROS injury rather than to direct photochemical damage.

First, the temperature-dependence reported by the authors themselves: treatments at 4 °C did not generate phototoxicity, whereas pre-incubation of cells at 42 °C followed by laser treatment led to significant phototoxicity at lower doses (1.5 W versus 2 W). The authors explicitly state that the rise in surface temperature appears to be critical for laser phototoxicity and further observe that cooling the tissue extended the window of safety. These are temperature-modulation effects, not photochemistry-modulation effects, and they are difficult to reconcile with a purely photochemical mechanism. The in vivo experimental design itself is explicitly thermal: the authors describe the mice as “dynamically treated” with the laser to maintain a target surface temperature of 45 °C, with the beam scanned in real time on the basis of thermal-camera feedback (Khan et al. 2015 [9], Figure 1).

Direct evidence of these dynamics appears in the authors’ own supplementary video, which embeds a “Maximum Skin Temperature” readout (Khan et al. 2015 [9], Supplementary Video). For the 55 °C condition, with laser activation occurring at approximately 8 s into the recorded timeline, peak skin temperatures during the active 30 s exposure period repeatedly reach approximately 60 °C, with the highest excursions touching ~62 °C, oscillating around the authors’ stated 55 °C target. A clear upward drift is visible across the exposure window, indicating that the feedback-controlled scanning protocol does not strictly maintain a stable target temperature but rather permits a slow accumulation of thermal dose. These peak excursions lie well above the 45 °C threshold the authors themselves identify and sit squarely within the temperature range at which sustained exposure produces protein denaturation and irreversible thermal injury per the Arrhenius framework.

Second, the beam-profile evidence is visible in the authors’ own published material. The in vivo exposure delivered 3.2 W of optical power across a 2 cm-diameter spot, corresponding to a nominal average irradiance of approximately 1 W/cm^2^. The thermal-camera image presented in the main text (Khan et al. 2015 [9], Figure 1) revealed, however, that the actual energy distribution was markedly concentrated at the beam centre—a rotationally symmetric, bulls-eye-shaped Gaussian peak characteristic of an uncorrected multimode diode source, with centre-of-beam irradiance substantially exceeding the nominal average. This is the beam-shape identified in the three-tier framework previously published by the present authors [19], in which focal thermal deposition at the Gaussian peak becomes the dominant mode of energy delivery to tissue. The supplementary video shows the beam moved in a small-amplitude orbital pattern around a more or less fixed central hub—distinct from a linear scanning motion, which would spread the dose across a larger tissue area—such that the Gaussian peak remains substantially co-located on the same tissue region throughout the exposure.

Direct visual evidence of this spatial non-uniformity can be seen in the clinical and histological images of Khan et al.’s Figure 1. In both the 45 °C and 55 °C conditions, tissue damage is concentrated centrally within the treated area rather than distributed uniformly across it; the damaged region expands with increasing target temperature (Figure 1c versus 1b) but in both cases remains a sub-region of the marked treatment circle. This spatial pattern is consistent with focal thermal deposition at the Gaussian peak, with the damage zone expanding as more of the beam’s spatial distribution crosses the thermal damage threshold at higher delivered power. A truly uniform irradiation at the nominal 1 W/cm^2^ spatial average would not be expected to produce this central-concentration pattern.

It should further be noted that accepted tissue optics analysis predicts a sub-surface fluence rate exceeding the incident surface fluence rate in the buildup region of the first 1–1.5 mm of skin, due to backscatter from forward-scattered photons (Welch & van Gemert 1995) [23]. The surface temperatures documented by Khan et al.’s thermal camera therefore represent an underestimate of the temperatures attained at the dermal-epidermal boundary, where the histological damage in Figure 1 of Khan et al. [9] is concentrated. The recorded peak surface temperatures of approximately 60–62 °C during the 55 °C exposure condition are accordingly consistent with sub-surface temperatures somewhat higher still at the depth of observed tissue injury.

Third, the authors’ control experiment using a 45 °C metal plate did not reproduce the phototoxicity (Khan et al. 2015 [9], Figure 1e), which they took as evidence against a purely thermal mechanism. We would note that static contact at the threshold temperature is not equivalent to the focal Gaussian-beam-centre temperatures achieved during photoexcitation, which would be expected to exceed 45 °C at the peak and to deposit additional energy beyond the surface. The metal-plate experiment thus rules out one specific form of thermal injury (uniform surface heating at threshold contact) but does not rule out focal thermal injury at higher centre-of-beam temperatures.

Fourth, the same authors’ subsequent publication on photobiomodulation-induced effects in burn wound healing [20] is consistent with a photothermal mechanism operating in this irradiance regime in their hands.

Taken together, the temperature dependence of phototoxicity, the Gaussian beam profile, the orbital scanning pattern around a near-static hub, and the protective effect of cooling collectively support a situation in which the observed injury reflects focal thermal damage at the Gaussian beam centre, with secondary ROS-mediated injury via inhibition of antioxidant enzymes. This is consistent with the broader principle, developed elsewhere in this manuscript, that high irradiance delivered over short timescales tends to produce photothermal effects, while lower irradiance delivered over longer timescales produces the photobiological responses characteristic of therapeutic PBM.

### 4.1. A Systematic Dosimetric Confound

The five studies examined share a common and previously unreported methodological flaw: each used an unrectified Gaussian source and calculated fluence by dividing the total power by total aperture area, implicitly assuming a flat-top uniform beam profile. This assumption is physically incorrect for semiconductor diode lasers and produces systematic underestimation of the actual energy delivered to the beam centre.

This discrepancy arises in unrectified Gaussian sources, where calculating dose from total aperture area understates the central irradiance by several-fold (≈2–4×). In four of the five studies, the corrected irradiance reaching the target places the exposure within the photothermal rather than the photobiological domain; the exception is Rhee et al., whose delivered dose lay within the therapeutic window and whose apparent tumour-promoting result is attributable to the immunodeficient host rather than to the dose (Section 3.1).

### 4.2. Device Category as a Fundamental Confound

Every device used in the five studies was a semiconductor diode source delivering an unrectified beam. The claim made here is that the output of such a source is spatially non-uniform with a pronounced central maximum, not that it conforms to any particular analytic profile. Edge-emitting diodes are typically astigmatic and elliptical, approximately Gaussian in the fast axis and frequently multimode in the slow axis, and broad-area emitters depart further still. The Gaussian description used throughout this manuscript is therefore a modelling convenience, adopted because it is tractable and because it is the profile most often assumed by default in the dosimetry literature. The argument does not depend on it. For any spatially non-uniform distribution whatever, the irradiance on the beam axis exceeds the aperture average, and it is that inequality, not its magnitude under a particular model, that the reappraisal turns on.

It should be stated directly that we have measured the spatial profile of none of the devices used in these studies, and that none of the original reports supplies one. The inference from device class to beam non-uniformity is drawn from the construction of diode sources rather than from measurement of these particular units, and it is graded accordingly in Section 3 and recorded among the limitations at Section 4.9.

The distinction between surgical and therapeutic devices is nonetheless real at the level of intended operating regime. Surgical diode lasers are engineered to concentrate power at the beam centre in order to achieve the coagulation and cutting for which they are clinically intended, and using one to characterise the biological effects of photobiomodulation is methodologically not ideal to study wound healing.

The distinction should not, however, be overstated, and in one respect it is more apparent than real. A device sold for photobiomodulation is not thereby beam-corrected. Very few current instruments incorporate genuine homogenisation, and those that do generally achieve it through a flat-top adaptor that is not in routine use. The commoner arrangements are partial: reduction in the central peak without redistribution of the energy removed from it, curved output surfaces that flatten the near-field appearance of the profile without homogenising it, and long-focal-length optics that produce an approximately uniform distribution at one working distance and at no other. Each of these improves the profile in a plane, and none of them makes the delivered distribution uniform in tissue. The comparison between homogenised and unrectified delivery is not a matter of inference: it has been made experimentally in tissue-simulating and ex vivo models, in which matched power delivered through a flat-top profile produces a broader, flatter and deeper zone of thermal effect than the same power delivered through an unrectified beam, which concentrates its effect at the axis [38,39,40]. The consequence for the present appraisal is that the device-category objection identifies the most severe cases rather than exhausting them, and a study is not exempted from the peak-to-average problem by having used an instrument marketed for photobiomodulation.

One further objection should be met here, since it is the obvious one. Optical coherence is unlikely to survive more than about a millimetre of tissue, and it might be argued on that basis that the character of the source ceases to matter almost immediately. It does not follow. The peak-to-average discrepancy is a radiometric and geometric property of the incident distribution rather than a coherence phenomenon, and the surface and near-surface irradiance at which thermal effects are initiated is established before any loss of coherence becomes relevant. The exposures reappraised here are characterised at the tissue surface, where the profile is at its least degraded and where the temperatures reported by Khan et al. were in fact measured.

A therapeutic device category adequate to the requirements of the field would be characterised by a factory-calibrated and beam-shaped output designed to produce a uniform irradiance across the treatment aperture, by a reported spatial power distribution rather than an assumed one and by operation below approximately 500 mW/cm^2^ at the tissue surface. On the evidence available in their reports, none of the five devices critiqued here satisfies all three, and none of the five reports supplies the measurement that would allow the first two to be assessed at all.

### 4.3. Mechanistic Reinterpretation—From Photothermal Artefacts to Immunological Opportunity

The biological findings in the studies that employed supra-therapeutic exposures are mechanistically consistent with photothermal overheating rather than with photobiological PBM effects. In those studies, the corrected irradiances and fluences lie well outside the photobiological window for PBM, in the range associated with photothermal tissue damage, protein denaturation, and cellular necrosis. Rhee et al. is a distinct case: the delivered dose was within the therapeutic window, and the apparent promotion is attributable not to thermal injury but to the absence of immune surveillance in the immunodeficient mice (below).

The ATF-4 orchestrated ER stress response identified by Khan et al., the tumour growth reported by Frigo et al. (immunocompetent BALB/c, supra-therapeutic dose), and the carcinogen-associated squamous cell carcinoma progression observed by de C Monteiro et al. are each mechanistically explained as responses to thermal injury rather than as photobiological stimulation of viable tissue. The proliferation reported by Rhee et al. (immunodeficient nude-mouse xenograft) is explicable on a different basis, set out below: a therapeutic-dose exposure in a host lacking the adaptive immunity through which PBM exerts its net anti-tumour effect.

Chen et al. demonstrated experimentally that irradiance—independently of total fluence—is the critical determinant of the biological response at the cellular level [41]. At the corrected irradiance values derived from the five critiqued studies, the biological response crosses from the photobiological into the photothermal domain regardless of exposure duration.

The apparent tumour growth observed in the Rhee and Frigo studies warrants specific attention, as these two papers carry the greatest weight in the safety literature as in vivo tumour models. In both cases the finding is more apparent than real, for compounding reasons that are independent of the dosimetric errors already identified. In the Rhee study, the host model is a nude mouse lacking functional T cells—the adaptive immune mechanisms that the two-tier model (see below) identifies as the primary route of PBM’s oncological benefit are absent by design. The delivered dose (100 mW/cm^2^, 15–30 J/cm^2^) lay within the therapeutic PBM window, so the proliferative and angiogenic response cannot be attributed to thermal injury; rather, it represents the local pro-proliferative and pro-angiogenic signalling that PBM can elicit, here left unopposed by the systemic, T-cell-mediated anti-tumour arm that an intact immune system would supply. The HIF-1α/Akt activation and TGF-β1 suppression Rhee et al. observed are consistent with this unmasked local response in an immunodeficient host. In the Frigo study, there may be an additional biological confounding factor: murine B16F10 melanoma cells sometimes have a high melanin content, and melanin absorbs strongly at 660 nm, amplifying photothermal effects in these cells substantially relative to normal tissue [37]. The experiment was therefore measuring the response of a uniquely photothermal-sensitive cell line to a surgical-range dental laser in an immunocompetent, allogeneic-mismatched host—a combination that is the least representative possible model for the clinical context of therapeutic PBM in oncological supportive care, where the target tissues are oral mucosa, skin, lymphatics, and vasculature, not melanin-rich melanoma cells. In Frigo, the tumour response is the predictable consequence of thermal stress at supra-therapeutic dose; in Rhee, it is the consequence of therapeutic-dose PBM acting in a host stripped of immune surveillance. Neither is evidence of photobiological tumour stimulation by therapeutic PBM in a clinically representative setting.

Khan et al. conducted mutagenicity assessments using the Ames test and plasmid cleavage assay, finding no evidence of mutagenic change or DNA strand breaks [9]. The absence of genotoxicity is consistent with the thermal interpretation and with the antioxidant inactivation mechanism described above: the cell injury pathway is ROS-mediated lipid peroxidation and ER stress caused by antioxidant enzyme inactivation, not direct genotoxic DNA base modification. This is mechanistically distinct from mutagenic photochemical damage and supports rather than undermines the safety argument for correctly dosed PBM at therapeutic parameters.

### 4.4. The Wider Worry-List—In Vitro Studies: Dosimetric Reappraisal

Beyond the five core studies examined in Section 3.1, Section 3.2, Section 3.3, Section 3.4 and Section 3.5, the oncological safety literature cites a number of in vitro cell culture studies and reviews. These studies share the fundamental methodological limitation identified in Section 2.5—a beam/well area mismatch and the absence of beam profile characterisation—and in several cases contain internal inconsistencies that undermine their conclusions. The two primary in vitro studies are examined here; the remaining citations (Navratil and Kymplova 2002; Al Ghamdi et al. 2012) are narrative or review publications and do not constitute primary experimental evidence [16,17].

Of these additional publications, the Sperandio et al. (2013) paper warrants specific dosimetric attention in the present context [15]. The paper reports that PBM promotes an aggressive phenotype in oral dysplastic and cancer cell lines via Akt/mTOR/CyclinD1 upregulation, and its title has entered the safety literature as evidence of photobiological tumour stimulation. A detailed review of the methods and supplementary data reveals a fundamental internal inconsistency that undermines this interpretation. The 660 nm InGaAlP/780 nm GaAlAs delivery system (Twin Laser MMOptics, Sao Paulo, Brazil) was used with a contact probe tip of stated spot area 0.039 cm^2^ at 40 mW, delivering the stated fluences of 2.05–6.15 J/cm^2^. The calculated irradiance at the cell surface is 40 mW ÷ 0.039 cm^2^ = 1026 mW/cm^2^—substantially above the wavelength-dependent photothermal threshold established by Zein, Selting, and Hamblin [11]: approximately 300 mW/cm^2^ at 600–700 nm (660 nm condition: 3.4× above threshold) and approximately 750 mW/cm^2^ at 800–900 nm (780 nm condition, just below this band: 1.4× above the near-infrared threshold). The exposure duration is not stated in the paper, but back-calculation from the stated fluences gives 2.0–6.0 s at each condition. Both wavelengths were therefore operating above the photothermal threshold at all three fluences. Critically, their Supplementary viability table (Table S1)—which the paper’s title and main text do not address—reports statistically significant decreases in cell viability across all tested conditions in SCC9 (6/6 wavelength/fluence combinations, *p* = 0.00852–0.00079) and the majority of conditions in SCC25 by 72 h. The DOK dysplastic cell line shows no significant viability reduction at any condition. The paper’s title claims increased aggressiveness; however, the paper’s own supplementary data show the cancer cells are dying. The Akt/mTOR upregulation finding rests on qualitative, non-quantified, non-blinded immunofluorescence images in the supplementary material without densitometry, scale bars, or exposure controls. No beam calibration or temperature monitoring is reported. The data pattern—delayed (72 h) viability loss in transformed lines only, wavelength-dependent cytotoxicity, and supra-threshold irradiance—is consistent with cumulative photothermal stress rather than photobiological tumour stimulation. This paper should not be cited as evidence that correctly characterised therapeutic PBM promotes cancer cell aggressiveness.

A second study from the wider worry-list concerns Sroka et al. (1999), a multi-wavelength action-spectrum study of laser-induced effects on mitosis in normal and tumour cell lines [14]. This paper warrants particular attention because, unlike Sperandio, its experimental apparatus is methodologically well controlled—calibrated power meter measurement before and after each treatment, ±10% intensity uniformity in a computer-controlled illumination chamber, temperature stability documented at 37 ± 0.5 °C, blinded double-counting by two observers, speckle averaging via fibre shaking, and a direct coherent vs non-coherent comparison using a Xe arc lamp at 410 nm. These are not the methods typical of a careless study and should be acknowledged as such.

The critique that follows is interpretive, not experimental. The authors used two assay methods in parallel: a quantitative BrdU/ELISA proliferation assay (the gold-standard method for cellular proliferation) and a qualitative Orcein single-cell mitotic count by manual microscopy. The BrdU assay returned no significant change at 0 h, 3 h, or 24 h after GaAlAs 805 nm diode laser illumination across all cell lines tested (their Figure 1). The Orcein manual count—which Sroka et al. themselves describe in the Discussion as “time-consuming and subjective”—returned the positive findings on which the paper’s headline conclusions rest. The authors explicitly chose the subjective method over the objective one on the grounds that it “seemed to be more appropriate”. The reasoning is inverted: the objective gold-standard measurement is set aside in favour of the subjective one precisely because the subjective one delivers a positive finding. The downstream consequences are substantial. The action-spectrum figure (their Figure 4) is built upon a single fluence × single irradiance condition selected from the Orcein-positive results. The tumour-stimulation result in this paper derives from one bladder carcinoma cell line (J82) at specific wavelengths and one fluence range, while three other carcinoma lines (MCF7, U373MG, ZMK1) showed no stimulation. The synchronised C2 time course (Figure 2 of Khan et al. [9]) reached significance at only one time point (24 h, 4 J/cm^2^). The cytochrome a/a_3_ attribution was arrived at by action-spectrum correlation only, which the authors themselves remarked was speculative. Sroka 1999 [14] should not be cited as evidence that therapeutic PBM stimulates tumour cell proliferation. The paper’s own gold-standard internal control does not support that interpretation, and the positive result on which the interpretation rests derives from an assay the authors themselves described as subjective [14].

These two examples should be set against the contrasting case of a study in which dosimetry genuinely within the therapeutic-window was used and directly verified. Kalampouka et al. (2024) [25] exposed cancer (MCF7, A549) and non-cancer (MCF10A, IMR-90) cell lines to 734 nm light at 63 mJ/cm^2^—a fluence two to three orders of magnitude below those in the studies critiqued here, delivered at a power density of 0.05 mW/cm^2^ over 20 min per day for six days (LED array of NIR 734 nm Rebel LEDs—Luxeon Star LED, Alberta, Canada). Critically, the authors characterised the source with a calibrated spectrometer and power meter and placed an optical temperature probe at the centre of the well, confirming directly that no thermal change occurred in the media during or after exposure in accord with the dosimetric transparency this paper advocates.

Our interpretation is already supported by the two existing systematic reviews of PBM safety in cancer. de Pauli Paglioni et al. (2019) [12] reviewed 27 clinical studies of PBM used to prevent or manage cancer-treatment toxicity and concluded that its use does not give rise to any tumour safety issues. The larger WALT review (Bensadoun et al., 2020) [10] synthesised 67 studies—43 in vitro, 15 in vivo, and 9 clinical—and reported that, although the in vitro literature is internally conflicting, the apparent tumour-promoting results were not replicated across the majority of studies, while the in vivo and clinical evidence with follow-up showed PBM to be safe with respect to tumour growth and could even confer an overall-survival benefit. That review attributed the in vitro discrepancies to the “wide variety of PBM parameters” used, several of which fell outside the MASCC/ISOO therapeutic range. The present reappraisal supplies the mechanism those syntheses stopped short of naming: where an apparent in vitro promotion signal exists, it arises at the concentrated Gaussian beam centre at supra-threshold irradiance and therefore reflects a photothermal rather than a photobiological process.

To place this in context, 0.05 mW/cm^2^ is an irradiance roughly four orders of magnitude below the ~1000 mW/cm^2^ delivered in the supra-threshold in vitro studies critiqued above (Sperandio, Khan) [9,15]—the low fluence is reached only because the exposure is sustained over 20 min, and at this irradiance no photothermal mechanism is possible, as the authors’ direct temperature measurement confirmed. This is the clearest demonstration in the literature that irradiance, not fluence alone, determines whether the photobiological or the photothermal domain applies. At this verified therapeutic-window dose, the effect was in the opposite direction to tumour stimulation: NIR light selectively induced senescence (permanent proliferation arrest) in the cancer cell lines while sparing the non-cancer lines (*p* > 0.05). The authors explicitly reconciled their finding with the proliferation results reported elsewhere with reference to fluence, proposing a hormetic cancer-selective window above approximately 20 mJ/cm^2^ and below 300 mJ/cm^2^, where proliferation rather than growth arrest is observed—citing the same high-fluence in vitro studies (Hu et al. 500–2000 mJ/cm^2^; Magrini et al. 1000 mJ/cm^2^) [42,43] that feature in the tumour-stimulation literature. This independent observation supports the central contention of the present analysis: the apparent tumour-stimulation findings cluster at fluences above the therapeutic window, and the beam-characterisation errors documented here push the actual delivered fluence in the critiqued studies further into the supra-therapeutic regime than their nominal figures suggest. Notably, MCF7—which appears in Sroka (no stimulation), Magrini (proliferation at 1000 mJ/cm^2^ but not at 5 mJ/cm^2^) and Kalampouka (senescence at 63 mJ/cm^2^)—exhibits exactly the fluence-dependent response the dose-dependence argument predicts.

**Human Clinical Trial Evidence:**

The human clinical evidence is highly reassuring rather than worrying. Brandão et al. (2018) conducted a retrospective safety analysis of locally advanced oral squamous cell carcinoma patients who received PBM for oral mucositis prevention during chemoradiotherapy, finding no evidence of tumour stimulation, accelerated progression, or adverse oncological outcomes [44]. Kauark-Fontes, Migliorati, Epstein et al. (2022) reported an interim analysis of a randomised, double-blind clinical trial of extraoral PBM for prevention of oral and oropharyngeal mucositis in head and neck squamous cell carcinoma patients receiving radiotherapy, confirming no adverse oncological safety indication in a clinically vulnerable population where PBM was applied in close proximity to tumour-bearing tissue [45]. Barasch et al. (2020) used an orthotopic head and neck squamous cell carcinoma animal model—an immunocompetent host with clinically representative tumour biology, in direct contrast to the immunodeficient nude-mouse model used by Rhee et al.—and demonstrated no tumour-promoting effect of PBM at therapeutic parameters [46]. Taken together, these three studies provide a bridge between the clinical and preclinical evidence and the dosimetric analysis presented here and confirm the safety conclusion: i.e., that correctly dosed PBM does not stimulate tumour growth in clinically representative models or in human patients receiving active cancer treatment.

A further and older line of evidence points in the same direction. From 1997, Chen and colleagues developed laser immunotherapy, in which controlled near-infrared laser heating of a tumour—far from stimulating its growth—produced not only local destruction but a systemic, tumour-specific immune response, eradicating untreated distant metastases and conferring durable resistance to subsequent tumour re-challenge [47,48]. The induced immunity was shown to be transferable to naïve animals and to depend on intact host adaptive immunity, confirming a genuinely immunological rather than merely local effect. Notably, the investigators observed that the light-absorbing dye used to localise the thermal effect could be omitted where the tumour itself absorbed sufficient laser energy directly—identifying the fundamental interaction as one of controlled laser-tissue heating driving anti-tumour immunity. This established body of work, sustained through to human clinical trials, is difficult to reconcile with the proposition that laser irradiation of tumour-bearing tissue is intrinsically tumour-promoting; on the contrary, when delivered within a controlled thermal window it has repeatedly produced the opposite effects.

The same dosimetric pattern persists in the current literature and is not an artefact of the age of the studies reappraised here. The most recent in vivo report of tumour promotion at the time of writing, Seo et al. 2024 [49], is again an athymic nude mouse xenograft. Its light source is a nine-lamp light-emitting diode array described as 20 W at the device but measured at 40 mW at the target; no irradiance, field area or spot size is reported, and the stated fluence of 2.4 J/cm^2^ cannot be reconstructed from the reported power, the aperture of the named meter and the stated exposure time by any route. Under the parameter-validity check specified in the MASCC/ISOO methodology, which requires that irradiance, fluence, and time per site reconcile, the report would not qualify for analysis [3].

### 4.5. The Immunological Dimension—A Two-Tier Model

The material in this subsection arises from the wider literature rather than from the dosimetric reappraisal above and is offered as a framework for prospective testing rather than as a finding of the present analysis.

If the exposures examined in Section 3 fall outside the therapeutic window, the question of what correctly dosed PBM produces in an oncological setting remains open. A two-tier framework offers one way to structure it. In a lower-tier regime, at therapeutic irradiances below the photothermal threshold, the primary event is mitochondrial photostimulation via cytochrome c oxidase [50], and any anti-tumour effect would be expected to operate through systemic immunomodulation: activation of superficial dermal vasculature and natural killer cells, engagement of the cutaneous neuroendocrine axis, and metabolic rescue of tumour-infiltrating CD8^+^ T cells whose effector function has been suppressed by the tumour microenvironment [51,52,53,54]. A survival benefit inconsistent with local mucosal effects alone, reported as a secondary observation in a Phase 3 mucositis trial by Antunes et al. [55], would be consistent with such a systemic mechanism but does not establish it.

In a higher-tier regime, at greater therapeutic fluences still below the photothermal threshold, a distinct pathway may become available by analogy with the systemic anti-tumour immunity produced by appropriately fractionated radiotherapy: sub-lethal mitochondrial perturbation releasing mitochondrial DNA to the cytosol, detection by cGAS, and STING-dependent cross-priming of cytotoxic T lymphocytes [56,57,58,59,60,61]. Whether PBM engages this pathway has not been demonstrated. The first supporting evidence in a syngeneic model was reported by Banstola et al. [62], in whom low-level PBM alone had no anti-tumour effect but restored oxidative phosphorylation in exhausted CD8^+^ T cells, and PBM combined with a STING agonist produced local tumour eradication. This is a single study in one model and is cited as motivation for testing, not as confirmation.

Older clinical observations are consistent with a systemic mechanism without establishing one. Mikhailov et al. reported, in a 10-year study of perioperative diode-laser treatment in breast cancer, both a survival difference relative to surgery-alone controls and—of more mechanistic interest—lymphoid-histiocytic infiltration in non-irradiated tumour parenchyma, together with histological improvement rather than stimulation at directly irradiated fluences of 0.24–1.53 J/cm^2^ [63]. The survival figures are tabulated in Supplementary Table S2. We note this study anticipates the language of immune activation later formalised in the radiotherapy abscopal literature, while cautioning that it is a single-centre study of its period and that, as recorded in Supplementary Table S1b, one systematic review classifies the same report as showing tumour promotion where another describes tumour regression—a discordance that illustrates why it cannot be treated as settled evidence in either direction.

### 4.6. The Warburg Metabolic Differential

The following is likewise offered as a mechanistic hypothesis rather than as an established route. A candidate basis for tumour selectivity is the differential metabolic state of tumour cells. Cells carrying out aerobic glycolysis in preference to oxidative phosphorylation (the Warburg effect) [64] both downregulate the cytochrome c oxidase on which PBM photostimulation depends and retain diminished antioxidant reserve. The same distinction—functional versus suppressed OXPHOS—would separate metabolically exhausted immune effector cells, which retain the regulatory architecture for OXPHOS reactivation, from Warburg-phenotype tumour cells, which do not. Kalampouka et al. provide in vitro support, reporting that therapeutic-window near-infrared light selectively induced senescence in cancer cell lines while sparing non-cancer lines, attributed to the elevated baseline reactive oxygen species and suppressed OXPHOS of the Warburg state [25].

One objection must be addressed, since it bears on the interpretation of the studies critiqued in Section 3. Thermal stress induces two independent cytoprotective systems, which must not be conflated. The heat shock protein chaperone system is transcription-dependent, has a latency period that underlies preconditioning protocols, and is not tumour-selective—many tumours constitutively over-express HSP70, making them better defended than normal tissue [65,66,67,68]. It therefore cannot account for selective tumour vulnerability, and we do not invoke it for that purpose.

The relevant distinction lies instead in the antioxidant and redox network. Khan, Tang, and Arany’s own supplementary data (their Supplementary Figure S3h,i) show that purified catalase and glutathione reductase lost activity following phototoxic dose in strongly absorbing but not in weakly absorbing conditions, with no temperature attached to either. That loss is not thermal denaturation: glutathione reductase activity in human erythrocytes is unaffected by heat stress across the physiological and mildly hyperthermic range [33], and the holo Cu/Zn-SOD1 enzyme retains its fold well above that range on even the most conservative reported values [31,32], so neither is disabled by any temperature attainable in these protocols. The authors’ own controls locate the mechanism on the oxidative side—helium substitution abolished phototoxicity while temperature rose unchanged, a metal probe at 45 °C produced no damage, cells at 4 °C were protected, and pre-incubation at 42 °C lowered the phototoxic threshold from 2 W to 1.5 W. Temperature lowers the dose at which oxidative disabling of the scavenger enzymes occurs, and marks the regime within which injury arises, without being its proximate cause.

Selectivity does not lie in differential susceptibility to that insult, which reaches both cell types, but in the reserve each retains. Mitochondrial MnSOD is downregulated in the majority of cancer types and catalase activity is reduced in many tumours relative to their tissues of origin; in a normal cell MnSOD remains as an antioxidant backstop, whereas in the Warburg tumour cell it has been constitutively silenced. The argument rests on this constitutive antioxidant deficit and does not depend on the debated proposal of locally elevated mitochondrial temperatures.

We record that this corrects a reading we ourselves have previously published, in Cronshaw, Parker, and Arany (2019) [69] and again in Cronshaw, Parker, Lynch, and Grootveld (2023) [19], in which the same enzyme-activity data were described as thermal inactivation at a specified temperature. That description originates in Khan et al.’s own discussion, referenced there to three sources, none of which assigns a temperature to the inactivation of either enzyme, and it cannot be sustained.

This bears directly on the studies critiqued in Section 3. At the surface temperatures the authors recorded, reaching 71 °C (Khan et al.’s Supplementary Figure S8a), delivered as a single acute exposure without preconditioning latency, the irradiated tissue lies far above both the adaptive HSP window and the mild-hyperthermia band (40–43 °C) used to potentiate radiotherapy [70,71], in the domain of frank thermal coagulation. Khan’s attribution of phototoxicity to ATF-4-orchestrated endoplasmic reticulum stress is consistent with this reading: ATF-4 is the effector of the integrated stress response, and its engagement as a death signal at these temperatures indicates cells driven beyond the adaptive threshold rather than photobiologically stimulated.

### 4.7. Implications for Dosimetric Reporting Standards

The two-tier mechanistic model has direct implications for dosimetric reporting in PBM oncology research. The boundary between Tier 1 and Tier 2 effects is not defined by fluence alone, but by the combination of wavelength, irradiance, total fluence, pulse parameters, and beam geometry at the target tissue. None of the five critiqued studies reported these parameters with sufficient accuracy to determine which mechanistic tier was being engaged. Three of the five reported parameters that were consistent, after Gaussian beam correction, with photothermal damage rather than photobiological stimulation at either tier.

Future studies investigating PBM in oncology contexts should report, at minimum: Gaussian or rectified beam profile; irradiance at the target tissue corrected for beam geometry and tissue optical properties [19]. They need to report the optical spot size at the target level, total fluence with distinction between centre-zone and outer-zone values for non-rectified Gaussian sources, device category, and the suitability of the biological model including immune status of the animal host.

Full development of this framework is beyond the scope of the present dosimetric reappraisal, which is confined to establishing that the studies examined were dosed outside the therapeutic window

### 4.8. The Clinical Consequences of Gaussian Beam Mischaracterisation

The dosimetric errors documented here have had measurable clinical consequences. The widespread citation of the five critiqued studies as safety evidence has contributed to a field-wide culture of conservatism in PBM oncology applications. Bensadoun et al.’s 2020 systematic review [10] documented the resulting clinical challenge: underdosing at depth is a prevalent problem because clinicians applying cautious low fluences at the tissue surface are not delivering sufficient energy in the therapeutic range to target tissues located even a few millimetres below the surface.

The route by which these studies reached clinical practice is less direct than it appears, and the citation trace described in Section 2.1.1 makes it visible. The classifications on which the hazard reading rests are carried by the review literature: Bensadoun et al. [10]; Hamblin, Nelson, and Strahan [6]; and the WALT position paper of Robijns et al. [21] between them supply every promotion classification recorded in Supplementary Table S1b. The documents closest to the bedside do not. Sonis et al. [5] argue from signalling pathways rather than from tumour outcomes, and cite two in vivo studies only, in a single clause describing the available investigations as limited and contradictory. The MASCC/ISOO clinical practice guideline of Zadik et al. [3] cites no primary in vivo tumour study at all; its caution against irradiating over a tumour site rests on one supporting reference, which is Sonis et al. The umbrella review and national-society recommendations of Grzech-Leśniak et al. [2] likewise cite no primary in vivo tumour study, report that a review of studies involving more than a thousand patients found no association between photobiomodulation and carcinogenicity or tumour promotion and record the avoidance of active tumour sites as carried forward from the WALT position paper notwithstanding the absence of evidence indicating tumour promotion.

Two qualifications belong with that observation. Neither guideline document is a safety review of the animal literature, and the absence of a tabulated animal evidence base is not in itself a deficiency in a document whose purpose is to recommend mucositis protocols. The trace also records what each document cites, which is not the same as what its authors read. What follows is nonetheless narrow and secure: the caution reaches clinical practice as an inherited provision rather than as a conclusion drawn from the studies reappraised here, while those studies supply its apparent evidential backing in the secondary literature surrounding it. This appraisal does not therefore overturn a guideline. It addresses the body of primary evidence commonly assumed to underwrite one and finds that assumption poorly founded.

The clinical consequence of this conservatism has been systematic under-dosing at depth. At 810 nm in tissue, depth attenuation requires approximately 4× the surface fluence to achieve therapeutic-range energy at 1 cm depth. A clinician applying a conservative surface fluence of 4 J/cm^2^ in response to inflated safety concerns is delivering approximately 1 J/cm^2^ to tissue at 1 cm—well below the biostimulatory threshold. The clinical lack of efficacy that results is sometimes misattributed to PBM itself being ineffective rather than to dosimetric inadequacy.

Systemic delivery routes for photobiomodulation exist, including transcutaneous and intravascular blood irradiation and point-source laser delivery at neurovascular sites, and the associated literature reports systemic anti-inflammatory and metabolic effects rather than pro-inflammatory ones [51,52,72,73]. These routes are not examined here and are noted only to indicate that the systemic effects reported for therapeutic-parameter PBM run contrary to a tumour-promoting mechanism.

### 4.9. Approaches to Beam Homogenisation, and Thermal Monitoring

The dosimetric problem identified throughout this appraisal is not that Gaussian sources are unsuitable for photobiomodulation, but that dosimetry calculated by dividing total power across total aperture area does not describe what the tissue at the beam axis receives. Several approaches to reducing that discrepancy are available, and they differ in what they achieve, what they cost, and what they leave unaddressed. None is a complete solution, and the choice between them is an engineering question rather than a clinical one.

**Beam expansion and defocusing.** Increasing the working distance, or introducing a diverging element, enlarges the illuminated area and lowers the average irradiance. It is the simplest mitigation, requires no additional optics, and is available on any device. It does not, however, change the shape of the distribution. The ratio of peak to average irradiance is preserved, so defocusing reduces the magnitude of the central excursion without removing the non-uniformity that causes it. It also incurs an inverse-square loss in delivered power and, in clinical use, an ill-defined treatment area.

**Refractive beam shapers.** Aspheric two-element systems can convert a Gaussian input to a flat-top output with high efficiency and low loss, and the design principles are well established [40,74]. Performance is specified for a particular input beam diameter and a particular output plane, so the benefit is realised at a defined working distance and degrades away from it.

**Microlens arrays and diffractive optical elements.** These divide the incident beam into sub-apertures and superimpose them at a design plane [74,75,76], producing a genuine flat-top distribution with sharply defined edges and high transmission. Chirped microlens arrays reduce the periodic intensity structure that regular arrays can impose. The limitations are that alignment tolerance is tight, that performance depends on the coherence properties of the source, and that uniformity is specified at the design plane only.

**Optical fibre delivery.** Mode scrambling within a multimode step-index fibre redistributes power across the core [74,77], so the near field at the fibre face approximates a top-hat rather than a Gaussian. This is one reason fibre-delivered systems are often better behaved than free-space diode output. The advantage is realised at or close to contact: beyond the fibre tip the output diverges according to the numerical aperture and the profile degrades with distance, so non-contact application recovers much of the original problem.

**Light-emitting diode arrays with diffusers.** Individual emitters are Lambertian rather than Gaussian and have low spatial coherence, so overlap between adjacent emitters together with a diffusing element can produce a quasi-uniform field. Microlens-based shaping of LED output has been analysed in detail. Uniformity depends on emitter pitch, diffuser characteristics and working distance, and adequate mixing is generally achieved only beyond a minimum separation from the array. LED delivery introduces its own dosimetric considerations in exchange: irradiance falls steeply with distance from the tissue, so contact and non-contact application are not equivalent, and an array-averaged irradiance can conceal a periodic spatial structure at short working distances.

**Scanning and galvanometric delivery.** Moving the beam across a defined area is an effective thermal mitigation, and the effect is measurable. In lean porcine muscle at 980 nm and an average irradiance of 1.0 W/cm^2^, a static Gaussian beam produced a surface rise of 12.0 °C at 60 s [19], while the same beam scanned across a 3 cm^2^ area produced 8.9 °C at 240 s; the corresponding flat-top values were 8.8 °C static and 5.3 °C scanning. The scanned conditions delivered 80 J/cm^2^ against 60 J/cm^2^ for the static ones, so a greater radiant exposure produced a smaller temperature rise.

Two qualifications should be attached. The mitigation operates by allowing thermal relaxation between passes, so its effectiveness depends on the scan rate relative to the thermal relaxation time [69] of the tissue, and systematic traversal of a defined area is not equivalent to movement about a fixed point. And scanning does not alter the instantaneous irradiance beneath the beam, nor does it correct the dosimetric calculation, which still rests on an aperture average. Scanning is therefore best understood as a mitigation of heat accumulation rather than as a substitute for beam characterisation.

**Flat-top handpiece delivery.** Homogenised delivery integrated into a treatment handpiece removes the peak-to-average discrepancy at the aperture, so that calculated and delivered irradiance coincide across the treatment field. Direct comparison has shown that where Gaussian delivery concentrates the biological effect at the beam centre, flat-top delivery distributes it across the aperture. In a matched-parameter comparison at 980 nm and 1.0 W/cm^2^ with collimated 1 cm^2^ applicators, the Gaussian device produced surface temperatures approximately 25% higher than the flat-top device (11.9 °C against 8.8 °C at 60 s, *p* = 0.022).

**Active cooling.** Heat may be removed as well as avoided or monitored. Surface cooling applied during or immediately after exposure lowers peak tissue temperature without altering the incident optical field [78], and therefore acts independently of beam profile, which makes it complementary to every approach above rather than an alternative to any of them. Khan et al. applied cryogen spray cooling to the treated area in vivo and abolished both the damaged area and the erythema score following an exposure that otherwise produced substantial injury, which is a demonstration from within the criticised study that the damage was temperature-dependent; read alongside their helium substitution, which abolished phototoxicity while temperature rose unchanged, the two controls delimit both factors of a two-factor mechanism. Preliminary work by the present authors has examined surface cooling in combination with surface thermometry, though this has not been evaluated in a controlled clinical trial and is mentioned here only as an indication of feasibility. Cooling carries its own considerations. It alters tissue optical properties during the exposure it is intended to protect. Its effect is limited in depth by thermal diffusion, so it protects the surface preferentially. And by suppressing the nociceptive response, it may remove the patient feedback that would otherwise terminate an excessive exposure, which is a safety consideration rather than a technical one and argues for pairing cooling with thermometry rather than substituting it.

A qualification applies to every approach in this list and should be stated precisely, because it is easy to state too strongly. Homogenisation acts on the incident distribution. It does not survive intact within tissue, where scattering redistributes photons within a few hundred micrometres of entry and progressively degrades the incident profile with depth [79], so no homogenisation technique should be represented as delivering a uniform exposure throughout an irradiated volume.

It does not follow that the benefit is confined to the surface, and the published comparisons indicate otherwise. In ex vivo and tissue-simulating models, the zone of thermal effect produced by a homogenised beam is broader, flatter and, over the depths these models resolve, deeper than that produced by an unrectified beam of the same power, which concentrates its effect on a narrow axis [38,39]. The distinction that matters is therefore between the profile, which is degraded by scattering within the first millimetre, and the distribution of delivered energy, which continues to reflect the geometry of the incident beam at depths well beyond the point at which the profile itself has been lost. Homogenisation addresses the surface dosimetry problem identified in the studies reappraised here, and it also alters the volume over which energy is deposited.

**Thermal monitoring.** Independently of beam profile, surface thermometry converts a calculated quantity into a measured one. Thermal imaging during treatment provides continuous confirmation that an exposure remains within the photobiological domain without relying on any assumption about the spatial distribution of the beam, and it is the single measurement that would have identified the conditions in three of the five studies reappraised here.

Two temperature figures are relevant here, and they derive from different sources and should be distinguished. TRPV1 nociceptive receptors activate at approximately 43 °C [80], a figure from the thermal nociception literature, which places the onset of thermally mediated discomfort. Separately, Khan et al. established 45 °C as the surface temperature at which phototoxic injury began in their model. The two figures are close but independent, and neither is derived from the other. A clinical cut-off of 45 °C for high-power applicators is therefore consistent with both the nociceptive threshold and the experimentally determined phototoxicity onset, rather than being derived from either alone.

### 4.10. Limitations

The constraints on this appraisal are set out here rather than distributed through the text, and several of them bear directly on how much weight its conclusions can carry.

**The reanalysis is retrospective and in part modelled.** None of the devices used in the studies examined was available for characterisation, and no beam profile was measured by us or reported by the original authors. Where central irradiance is estimated, it is estimated from an assumed profile, and those estimates are presented as ranges and graded accordingly in each study’s certainty table. A distinction should be drawn between the direction and the magnitude of the resulting correction. For any spatially non-uniform distribution, the irradiance on the beam axis necessarily exceeds the aperture average, so the underestimation of central irradiance in these studies follows without any assumption about profile; the factor by which it was underestimated does not, and rests on the model described in Section 2.2.

**Thermometry is absent from most of the studies examined.** Only Khan et al. 2015 [9] report tissue temperature. For the remaining studies, any thermal contribution to the reported findings is inferred from irradiance, absorption, and duration rather than measured, and alternative accounts that do not invoke temperature cannot be excluded from the published data. This is most consequential for de C Monteiro et al., discussed below.

**The selection of studies is traceable but not systematic.** The identification procedure is set out in Section 2.1 and can be repeated, but it is not a systematic review, and the manuscript does not claim to characterise the whole of the evidence base on photobiomodulation safety in oncology. Two of the studies examined were not identified through the screened collection and were included on independent grounds, which Section 2.1 states explicitly. The screened collection is itself a curated private database rather than a database search, and its outcome tags were assigned within the author group; it is reported as a denominator and cross-check and not as a reproducible search strategy.

**Not every study is disposed of with equal confidence.** The certainty grading applied in Section 3 is intended to make this visible rather than to obscure it, and several elements central to our reading are graded at the weakest tier. de C Monteiro et al. in particular does not fail on host model or device category, records no temperature, and is set aside on the convergence of three moderate arguments rather than on any decisive one; Section 3.3.6 states the alternative reading that the published data do not exclude.

**In vitro evidence is admitted here for limited purposes**, as set out in Section 2.2, and the mechanistic propositions advanced in Section 4 rest in part on it. Those propositions are offered as hypotheses for prospective testing and are labelled as such; they are not established by the reappraisal presented here, which is dosimetric.

**Several authors have declared commercial interests** in photobiomodulation, as recorded in the Conflicts of Interest statement. The analytical criteria applied in Section 2.2 were defined before any recalculation was undertaken and applied uniformly to every study examined, including those whose findings are consistent with our argument, and Section 4.8 surveys the available approaches to beam homogenisation rather than advocating any one of them. The declared interests are noted here for transparency in relation to the discussion of beam delivery in Section 4.9, where a range of approaches is surveyed rather than any one recommended.

**Finally, and most importantly, the argument of this manuscript is negative and bounded.** It establishes that a specific body of frequently cited preclinical evidence does not securely demonstrate the hazard attributed to it. It does not establish that photobiomodulation is oncologically safe. Studies that were not competent to demonstrate a hazard are equally not competent to demonstrate its absence, and no inference about safety in any particular clinical context should be drawn from their reappraisal. That question requires prospective evidence obtained with characterised beam delivery, reported spatial power distribution and documented thermal monitoring, and it remains open.

## 5. Conclusions

Five studies that are frequently cited as experimental evidence of an oncological hazard associated with photobiomodulation have been reappraised against their own reported dosimetry.

In four of the five, the exposures delivered lay outside the therapeutic photobiological window on the authors’ own stated parameters, before any correction for beam profile is applied. Khan, Tang, and Arany recorded surface temperatures of 55 °C and, in places, 71 °C, and describe injury above 55 °C extending to coagulation, necrosis, contracture, paralysis, and death. Frigo et al., across two studies, delivered 2.5 W/cm^2^, approximately 25 times the irradiance conventionally applied in clinical protocols, and observed promotion only once accumulated exposure crossed a threshold lying between 150 and 450 J/cm^2^. de C Monteiro et al. applied an irradiance and a per-session fluence that their own discussion describes as high.

The fifth, Rhee et al., is the exception. Its dosimetry lies within the therapeutic range, which none of the other four does. What limits the inference drawn from it is the host: an athymic animal without functional T lymphocytes cannot express an immune-mediated response, and a study conducted on one measures only the local component of a two-sided process.

Three conclusions follow, and they are bounded.

None of these studies provides dosimetrically secure evidence that photobiomodulation delivered within the therapeutic range to an immunocompetent, clinically representative model stimulates tumour growth. The exposures examined are not those used in clinical care, and findings obtained outside a therapeutic window do not characterise behaviour within it.

Spatial power distribution should be reported as a matter of course in photobiomodulation research. None of the studies examined here measured it, and in each case the dosimetry rests on an aperture average that cannot describe what the tissue on the beam axis received. Reporting irradiance and fluence without the profile and geometry that generated them leaves a study unreproducible in the respect that matters most for safety.

A distinction should be drawn in closing. This appraisal establishes that a specific and frequently cited body of preclinical evidence does not securely demonstrate the hazard attributed to it. It does not establish that photobiomodulation is oncologically safe. Studies that were not competent to demonstrate a hazard are equally not competent to demonstrate its absence, and no conclusion about safety in any particular clinical context should be drawn from their reappraisal. That question requires prospective evidence obtained with characterised beam delivery, reported spatial power distribution, and documented thermal monitoring, and it remains open.

### Future Perspectives

The following arises from the wider literature rather than from the reappraisal presented above and is offered as a framework for prospective testing rather than as a conclusion of this work.

If the exposures examined here fall outside the therapeutic window, the question of what correctly dosed photobiomodulation does in an oncological setting remains unanswered rather than settled. A two-tier framework may be a useful way to structure that question: a lower-irradiance regime in which immunomodulatory effects predominate, and a higher-irradiance regime, still short of photothermal injury, in which effects on lymphocyte metabolism and innate immune signalling may become relevant. Whether either tier confers benefit, harm, or neither is not established by the present analysis and cannot be, since the studies reappraised here did not operate in either regime.

What such a test would require is now reasonably clear. An immunocompetent host, a syngeneic tumour model, characterised beam delivery with a reported spatial power distribution, thermometry throughout exposure, and doses selected to lie demonstrably within the therapeutic window rather than to exceed it. The two studies of Khan et al. examined here illustrate why the last of these matters: the same source, at the same wavelength, produced phototoxic injury at one irradiance and therapeutic benefit at another. Until studies of that design are available, the oncological safety of photobiomodulation will continue to rest on clinical experience and on the absence of signal in the human literature rather than on preclinical demonstration.

## Figures and Tables

**Table 1:** Certainty grading of the reappraisal of Rhee et al. 2016 [27].

Element	Grade	Basis
Reported dosimetry is internally consistent and lies within therapeutic range	**Verified**	Arithmetic, from the authors’ own stated values
Host is athymic and lacks functional T cells	**Verified**	Stated by the authors; a defining property of the strain
Estimated central irradiance remains sub-photothermal	**Strongly supported**	Follows for any non-uniform profile; magnitude modelled, not measured
Absence of adaptive immunity permits unopposed local signalling	**Plausible**	Consistent with the proposed mechanism; not demonstrated in this study
The same exposure in an immunocompetent host would not produce this result	**Plausible**	Consistent with the wider animal-tier pattern; not tested here

Bold indicates the certainty grade assigned to each element.

**Table 2:** Certainty grading of the reappraisal of Frigo et al. 2009 and 2018 [8,26].

Element	Grade	Basis
Stated parameters are internally consistent within and between the two studies	**Verified**	Recomputation; 3/9/21 J at 50 mW across 0.02 cm^2^ reproduces 150/450/1050 J/cm^2^ at 60/180/420 s
Stated irradiance is approximately 25× conventional therapeutic values	**Verified**	Comparison of stated values against published protocol ranges
A threshold lies between 150 and 450 J/cm^2^ at constant irradiance	**Verified**	The 2018 four-group design
Host is immunocompetent and the model allogeneic	**Verified**	Strain and cell line as reported
Axial irradiance exceeds the stated average	**Verified**	Necessary for any non-uniform distribution
Magnitude of that excess	**Plausible**	Modelled; neither device was characterised
Melanin absorption raises absorbed energy density in this target	**Strongly supported**	Established optical properties of melanised tissue at 660 nm
The threshold reflects thermal accumulation	**Plausible**	Consistent with Arrhenius behaviour; no thermometry was performed in either study
The same exposures within the therapeutic range would not promote growth	**Plausible**	Consistent with the 150 J/cm^2^ arm and the wider animal-tier pattern; not directly tested

Bold indicates the certainty grade assigned to each element.

**Table 3:** Certainty grading of the reappraisal of de C Monteiro et al. 2011 [29].

Element	Grade	Basis
Reported arithmetic is internally consistent	**Verified**	Recomputation from the authors’ stated values
Stated irradiance and fluence lie above conventional therapeutic ranges	**Verified**	Comparison of stated values against published protocol ranges
The power meter used cannot resolve spatial distribution	**Verified**	Instrument specification
Axial irradiance exceeds the stated aperture average	**Verified**	Necessary for any non-uniform distribution
Magnitude of that excess (approximately 0.8–1.7 W/cm^2^ central)	**Plausible**	Modelled from an assumed profile; device not characterised
The authors regarded their own parameters as high	**Verified**	Stated in their discussion
The reported effect is statistically fragile	**Verified**	*n* = 5 per group, *p* = 0.04, effect carried by two animals
Temperature contributed materially to the observed progression	**Plausible**	No thermometry was performed; inferred from irradiance alone
A therapeutic-range exposure would not have produced this result	**Plausible**	Consistent with the wider animal-tier pattern; not tested here

Bold indicates the certainty grade assigned to each element.

**Table 4:** Certainty grading of the reappraisal of Khan, Tang, and Arany 2015 [9].

Element	Grade	Basis
In vitro arithmetic is internally consistent; in vivo fluence is not determinable	**Verified**	Recomputation from stated values; total irradiation time and duty cycle unreported
Surface temperatures reached 55 °C and, in places, 71 °C	**Verified**	The authors’ own thermometry, their Supplementary Figure S8a
Central irradiance exceeds the aperture average	**Verified**	Necessary for any non-uniform distribution; the authors themselves assume Gaussian
Magnitude of that excess is approximately 2–4×	**Plausible**	Modelled from an assumed profile; the device was not characterised
Enzymes lost activity in absorbing but not clear-well conditions	**Verified**	The authors’ Supplementary Figure S3h,i
The effector is oxygen-dependent	**Verified**	The authors’ helium control
Temperature is sensitising rather than causal	**Strongly supported**	The authors’ 45 °C probe, 4 °C and 42 °C pre-incubation controls
ATF-4 induction is downstream of oxidative insult rather than a direct photobiological signal	**Strongly supported**	Integrated stress response literature; heat alone induces the same signature
Injury at 71 °C lies in the coagulation range	**Strongly supported**	Thermal damage literature; not assayed histologically at that temperature in this study
Coagulation and necrosis occur above 55 °C in vivo	**Verified**	The authors’ own histological description, their Supplementary Figure S1
Injury above 55 °C extended to paralysis and death	**Verified**	Khan et al. Supplementary Table S1
Apoptosis predominates in vitro at phototoxic dose	**Verified**	The authors’ TUNEL assay; note this is the in vitro regime, not the high-temperature in vivo one
The authors applied a Gaussian-based correction to their in vitro dosimetry	**Verified**	Their Supplementary Table S4 legend and the arithmetic of the reported values
The in vitro correction cannot be independently reconstructed	**Verified**	Derivation not given in the published record

Bold indicates the certainty grade assigned to each element.

**Table 5:** Certainty grading of Khan et al. 2021 [20] as an internal control.

Element	Grade	Basis
Per-session radiant exposure of 2.1 J/cm^2^ lies within the therapeutic range	**Verified**	Arithmetic from the authors’ stated values
The same device and wavelength were used in both studies	**Verified**	Stated by the authors in both papers
Irradiance differed by approximately 14-fold between the two studies	**Verified**	Comparison of the authors’ stated values
The 2021 exposure remains sub-photothermal at the beam centre	**Strongly supported**	Follows on any plausible profile; magnitude modelled, not measured
Irradiance rather than source identity accounts for the difference in outcome	**Strongly supported**	The two studies differ in irradiance while holding device, wavelength and group constant; other differences remain
Host immune status contributed to the difference in outcome	**Not assessed**	The models differ in indication as well as in host; no inference is drawn

Bold indicates the certainty grade assigned to each element.

## Data Availability

No new data were created or analysed in this study. Data sharing is not applicable to this article.

## Supplementary Materials

The following supporting information can be downloaded at: https://www.mdpi.com/article/10.3390/biomedicines14081859/s1. Supplementary Table S1, citation-trace record of the identification of candidate studies, comprising Table S1a (citing documents traced), Table S1b (citation matrix and study register), and Table S1c (contextual abstract screen); Supplementary Table S2, tabulated long-term survival data from Mikhailov et al. referenced in the Discussion. Supplementary Table S1b contains full citations for studies examined during the identification procedure; those not cited in the main text are listed there for transparency of the selection process.
