# Supplementary material for: A Dosimetric Reappraisal of the Photobiomodulation Literature Assessing Animal Safety and Phototoxicity Evidence for Photobiomodulation in Oncology: Phantom Dangers
Source: Biomedicines. 2026 Aug 19;14(8):1859. doi: 10.3390/biomedicines14081859 (PMC13510168; doi:10.3390/biomedicines14081859)
Supplement: Supplementary file 1 [file biomedicines-14-01859-s001.zip › biomedicines-4454416-supplementary.pdf]

Supplementary Materials

A Dosimetric Reappraisal of the Photobiomodulation Literature Assessing Animal Safety and Phototoxicity Evidence for Photobiomodulation in Oncology: Phantom Dangers

Cronshaw M, Parker S, Carroll JD, Epstein JB, Grzech-Leśniak K, Hamblin MR

Manuscript biomedicines-4454416

Supplementary Table S1. Citation-trace record of the identification of candidate studies

This record documents how the primary studies reappraised in Section 3 of the manuscript were identified. The manuscript concerns studies repeatedly cited as experimental evidence of an oncological hazard associated with photobiomodulation. That criterion was operationalised as citation tracing: a defined set of published reviews, position papers and guideline documents on photobiomodulation safety in oncology was read in full, and for each document the primary studies it cites and classifies as showing tumour promotion were recorded.

This is citation tracing, not a systematic review. No database search string was executed, no PRISMA flow applies, and no claim of exhaustive coverage of the primary literature is made. The output is a transparent record of which studies the field itself has repeatedly nominated as hazard evidence. Each citing document was traced by one author and the extracted classifications independently verified against the source document by a second author.

Inclusion rule. A primary study was carried forward for dosimetric reappraisal where it was classified as showing tumour promotion in two or more of the citing documents and was conducted in a tumour-bearing animal or chemical carcinogenesis model. The restriction to the animal tier is deliberate and is part of the rule: several in vitro reports meet or exceed the citation threshold and are addressed in Section 4.4 of the manuscript rather than in Section 3.

Studies analysed on other grounds. Two studies appraised in Section 3 were not identified by this procedure and are included on separate stated grounds: Khan, Tang and Arany 2015, the source of the 45 °C surface-temperature threshold subsequently applied as a safety ceiling, and Khan et al. 2021, an internal control using the same device and group at therapeutic irradiance. See Section 2.1.4 of the manuscript.

Declaration of overlap. One of the six documents traced, Grzech-Leśniak et al. 2025, is led by a co-author of the present manuscript. It contributes no promotion classifications to the count and therefore cannot have influenced the selection, but the overlap is declared here.

Table S1a. Citing documents traced

ID	Citing document	Type	Location of the hazard classification
D1	Bensadoun RJ, Epstein JB, Nair RG, Barasch A, Raber-Durlacher JE, Migliorati C, et al. Safety and efficacy of photobiomodulation therapy in oncology: a systematic review. Cancer Med. 2020;9(22):8279–8300.	Systematic review (safety)	Table 2: fifteen in vivo studies classified by outcome direction.
D2	Hamblin MR, Nelson ST, Strahan JR. Photobiomodulation and cancer: what is the truth? Photomed Laser Surg. 2018;36(5):241–245.	Narrative review (safety)	Section "Can PBM Stimulate Cancer?": three in vivo studies named; in vitro studies cited separately.
D3	Robijns J, Nair RG, Lodewijckx J, Arany P, Barasch A, Bjordal JM, et al. Photobiomodulation therapy in management of	Position paper / consensus guideline	Section 3.2.1, in vitro and in vivo safety data.

ID	Citing document	Type	Location of the hazard classification
	cancer therapy-induced side effects: WALT position paper 2022. Front Oncol. 2022;12:927685.		
D4	Sonis ST, Hashemi S, Epstein JB, Nair RG, Raber-Durlacher JE. Could the biological robustness of low level laser therapy (photobiomodulation) impact its use in the management of mucositis in head and neck cancer patients. Oral Oncol. 2016;54:7–14.	Narrative review / commentary	No study-level hazard classification. The argument is pathway-based; in vivo investigations are cited collectively in the Introduction as limited and contradictory.
D5	Zadik Y, Arany PR, Fregnani ER, Bossi P, Antunes HS, Bensadoun RJ, et al. Systematic review of photobiomodulation for the management of oral mucositis in cancer patients and clinical practice guidelines. Support Care Cancer. 2019;27(10):3969–3983.	MASCC/ISOO clinical practice guideline	Discussion, paragraph on potential long-term risk. No primary in vivo tumour study is cited; the single supporting reference is D4.
D6	Grzech-Leśniak K, Fiegler-Rudol J, Grzech-Leśniak Z, Matys J, Marek G, Skaba D, et al. Laser therapy for the management of oral mucositis: an umbrella review — official recommendations of the Polish Society for Laser Dentistry. Dent Med Probl. 2025;62(6):1157–1175.	Umbrella review / national society recommendations	Long-term safety section. No primary in vivo tumour study cited; records tumour-site avoidance as carried forward from D3 notwithstanding the absence of evidence of promotion.

Table S1b. Citation matrix and study register

Codes: P, classified as showing tumour promotion; I, classified as inhibitory, regressive or showing no enhancement; N, cited but not classified in either direction; P/I, classified as showing both directions at different doses; —, not cited. The count column records P classifications only.

D1 records the whole of the in vivo tabulation in Bensadoun et al., Table 2, so that the animal-tier denominator is visible. Of the fifteen in vivo studies tabulated there, three are classified as showing tumour promotion without qualification, two report opposite directions of effect at different doses, one reports promotion only at the highest of three doses, and nine report no promotion.

ID	Primary study	Tier	D1	D2	D3	D4	D5	D6	Count	Disposition
S01	Frigo et al. 2009	Animal	P	P	P	—	—	—	3	Appraised, Section 3.2
S02	Rhee et al. 2016	Animal	P	P	—	—	—	—	2	Appraised, Section 3.1
S03	de C Monteiro et al. 2011	Animal	P	P	P	N	—	—	3	Appraised, Section 3.3
S04	Frigo et al. 2018	Animal	P	—	—	—	—	—	1	Appraised with S01, Section 3.2
S05	Mikhailov et al. 1993	Animal	P/I	—	I	—	—	—	1	Excluded; biphasic, dosimetry not assessable
S06	Abe et al. 1993	Animal	P/I	—	—	—	—	—	1	Excluded; parameters not stated
S07	de C Monteiro et al. 2013	Animal	N	—	—	—	—	—	0	Appraised with S03 as companion report
S08	Myakishev-Rempel et al. 2012	Animal	I	—	I	N	—	—	0	No hazard classification
S09	Ottaviani et al. 2016	Animal	I	I	I	—	—	—	0	No hazard classification
S10	Barasch et al. 2020	Animal	N	—	I	—	—	—	0	No hazard classification
S11	Ulrich et al. 1996	Animal	N	—	—	—	—	—	0	No hazard classification
S12	Zhang et al. 2009	Animal	I	—	—	—	—	—	0	No hazard classification

ID	Primary study	Tier	D1	D2	D3	D4	D5	D6	Count	Disposition
S13	Wikramanayake et al. 2013	Animal	N	—	—	—	—	—	0	Not a tumour-growth endpoint
S14	Khori et al. 2016	Animal	I	—	—	—	—	—	0	No hazard classification
S15	Petrellis et al. 2017	Animal	I	—	—	—	—	—	0	No hazard classification
S16	Sperandio et al. 2013	In vitro	N	P	P	P	—	—	3	Outside eligible universe; Section 4.4
S17	Sroka et al. 1999	In vitro	N	P	P	—	—	—	2	Outside eligible universe; Section 4.4
S18	Gomes Henriques et al. 2014	In vitro	P	—	P	P	—	—	3	Outside eligible universe; Section 4.4
S19	Chu et al. 2010	In vitro	P	—	P	P	—	—	3	Outside eligible universe; Section 4.4
S20	AlGhamdi et al. 2012	In vitro	—	P	—	N	—	—	1	Outside eligible universe
S21	Khan, Tang and Arany 2015	Animal	—	—	—	—	—	—	0	Appraised, Section 3.4; identified outside the trace
S22	Khan et al. 2021	Animal	—	—	—	—	—	—	0	Appraised, Section 3.5; internal control
S23	Revazova et al. 2001	Animal	—	—	—	—	—	—	0	Contextual screen only; immunodeficient host
S24	Seo et al. 2024	Animal	—	—	—	—	—	—	0	Contextual screen only; noted in Section 4.4

Notes on individual records.

S04, Frigo et al. 2018. One promotion classification only, so the two-document rule is not met. It is appraised alongside Frigo et al. 2009 as a companion report from the same laboratory, and that basis is stated in Section 3.2. D1 records dose-dependent tumour growth at doses of 9 J and above, with no effect at 3 J.

S05, Mikhailov et al. 1993. D1 records tumour retardation and prolonged life span at 0.46 J/cm² together with increased tumour weight at 1.53 J/cm² in the same report; D3 cites the low-dose regression arm. The apparent discordance between the two documents reflects a biphasic result rather than a disagreement of fact. Irradiance, beam profile and exposure time are not reported, so the study fails the assessability criterion.

S06, Abe et al. 1993. D1 records inhibition when applied on the first day after implantation, by both direct and indirect routes, and enhancement only by the indirect route at fourteen days. Fluence is recorded as not stated, so independent recalculation is not possible.

S07, de C Monteiro et al. 2013. D1 classifies this report as showing no influence on tumour behaviour. On the primary record its laser-only arm produced poorly differentiated carcinoma in two of five animals against none in ten controls, with well-differentiated carcinoma significantly more frequent in the controls. The classification and the primary record diverge, and the study is appraised in Section 3.3 alongside its 2011 companion rather than relied upon as a null result.

S11 to S15. These five animal-tier records appear in the same tabulation as the promotion classifications and report no tumour promotion. They are listed so that the denominator against which the identified studies should be read is visible, and so that the identification rule can be seen to have been applied to the whole of the tabulation rather than to a selected part of it.

S23 and S24. Identified in the contextual abstract screen rather than by the citation trace. Both are immunodeficient-host studies reporting promotion and neither is cited as hazard evidence by any traced document. They are recorded so that the animal-tier promotion reports known to the author group are visible in full.

Full citations for records in Table S1b

S01 Frigo L, Luppi JSS, Favero GM, Maria DA, Penna SC, Bjordal JM, et al. BMC Cancer. 2009;9:404. · S02 Rhee YH, Moon JH, Choi SH, Ahn JC. Photomed Laser Surg. 2016;34(6):229–35. · S03 de C Monteiro JS, Pinheiro AN, de Oliveira SC, Aciole GT, Sousa JA, Canguss MC, et al. Photomed Laser Surg. 2011;29(11):741–5. · S04 Frigo L, Cordeiro JM, Favero GM, et al. High doses of laser phototherapy can increase proliferation in melanoma stromal connective tissue. Lasers Med Sci. 2018;33(6):1215–23. · S05 Mikhailov VA, Skobelkin OK, Denisov IN, Frank GA, Voltchenko NN. Laser Ther. 1993;5(1):33–8. · S06 Abe M, Fujisawa K, Suzuki H, Sugimoto T, Kanno T. Role of 830 nm low reactive level laser on the growth of an implanted glioma in mice. Keio J Med. 1993;42(4):177–9. · S07 de C Monteiro JS, de Oliveira SC, Reis Junior JA, Gurgel CAS, de Souza SCOM, Pinheiro ALB, et al. Lasers Med Sci. 2013;28(3):1017–24.

S08 Myakishev-Rempel M, Stadler I, Brondon P, Axe DR, Friedman M, Nardia FB, et al. Photomed Laser Surg. 2012;30(9):551–8. · S09 Ottaviani G, Martinelli V, Rupel K, Caronni N, Naseem A, Zandonà L, et al. EBioMedicine. 2016;11:165–72. · S10 Barasch A, Li H, Rajasekhar VK, Raber-Durlacher J, Epstein JB, Carroll J, et al. Support Care Cancer. 2020;28(6):2721–7. · S11 Ulrich M, Jahn R, Zywietz F, Schäfer H, Jungblut KH. In: Waidelich W, Waidelich R, eds. Laser in der Medizin. Springer; 1996. · S12 Zhang L, Xiong Z, Li Z, Yao B, Zhang D. J Radiat Res. 2009;50(2):109–17. · S13 Wikramanayake TC, Villasante AC, Mauro LM, et al. Lasers Med Sci. 2013;28(3):701–6. · S14 Khori V, Alizadeh AM, Gheisary Z, et al. Lasers Med Sci. 2016;31(9):1775–82. · S15 Petrellis MC, Frigo L, Marcos RL, et al. J Photochem Photobiol B. 2017;177:69–75.

Table S1c. Contextual abstract screen

Reported in Section 2.1.2 of the manuscript as context and not as the identification method. The collection is a curated private database rather than the output of a reproducible search, and the outcome tags were assigned within the author group and cross-checked rather than independently duplicated.

Study-design tier	Records	Promoting	Inhibitory or protective	Null	Mixed, biphasic or not determinable
In vitro	146	26			
Human	82	0			30 not determinable
Animal	64	9			
Review	12				
Out of scope	13				
Unclassified	11				
Total	328	35	132	59	24 mixed or biphasic; 96 not determinable

Cells left blank were not resolved to tier level in the screen record.

Supplementary Table S2. Tabulated long-term survival data from Mikhailov et al. referenced in the Discus-sion

Referenced in Section 4.5 of the manuscript. Ten-year prospective study of perioperative gallium arsenide semiconductor diode laser treatment (890 nm, up to 5 W, up to 3000 Hz) in 41 patients with Stage II–III breast cancer against 40 surgery-alone controls. Treatment was administered before surgery and for two years postoperatively.

Outcome	Stage	Laser plus surgery	Surgery alone	Difference
Ten-year survival	II	86.9%	76.19%	+10.7 points
Ten-year survival	III	83.3%	68.4%	+14.9 points
Five-year survival	II	100%	85.71%	+14.3 points
Five-year survival	III	94.44%	78.94%	+15.5 points
Recurrence-free survival at ten years	II	82.6%	66.6%	+16.0 points

Outcome	Stage	Laser plus surgery	Surgery alone	Difference
Recurrence-free survival at ten years	III	77.7%	57.9%	+19.8 points

Postoperative complications were reduced by 15.3 percentage points. Two further findings are of mechanistic rather than clinical interest and are discussed in Section 4.5: lymphoid-histiocytic infiltration was reported in non-irradiated tumour parenchyma, and direct irradiation of tumours at 0.24–1.53 J/cm² improved the histological appearance by I–III degree rather than stimulating growth.

The five-year recurrence-free rates reported in the same paper were 91.3% for Stage II (about 77.7% in controls) and 82.35% for Stage III (60% in controls). The 82.35% figure is sometimes misread as the Stage II ten-year value; the source assigns it to Stage III at five years.

This is a single-centre study of its period and is cited as an observation consistent with a systemic immune mechanism rather than as establishing one. As recorded in Supplementary Table S1b, one systematic review classifies the same investigator group's experimental report as showing tumour promotion where another describes tumour regression.
